# DYRK3 phosphorylates SNAPIN to regulate axonal retrograde transport and neurotransmitter release

**DOI:** 10.1038/s41420-022-01290-0

**Published:** 2022-12-30

**Authors:** Ye Hyung Lee, Bo Kyoung Suh, Unghwi Lee, Seung Hyun Ryu, Sung Ryong Shin, Sunghoe Chang, Sang Ki Park, Kwang Chul Chung

**Affiliations:** 1 Department of Systems Biology, College of Life Science and Biotechnology, Yonsei University, Seoul, Korea; 2 Department of Life Sciences, Pohang University of Science and Technology, Pohang-si, Gyeongsangbuk-do Korea; 3 Department of Physiology and Biomedical Sciences, Seoul National University College of Medicine, Seoul, Korea

**Keywords:** Phosphorylation, Chemical modification

## Abstract

Among the five members of the dual-specificity tyrosine-phosphorylation-regulated kinase (DYRK) family, the cellular functions of DYRK3 have not been fully elucidated. Some studies have indicated limited physiological roles and substrates of DYRK3, including promotion of glioblastoma, requirement in influenza virus replication, and coupling of stress granule condensation with mammalian target of rapamycin complex 1 signaling. Here, we demonstrate that serum deprivation causes a decrease in intracellular DYRK3 levels via the proteolytic autophagy pathway, as well as the suppression of *DYRK3* gene expression. To further demonstrate how DYRK3 affects cell viability, especially in neurons, we used a yeast two-hybrid assay and identified multiple DYRK3-binding proteins, including SNAPIN, a SNARE-associated protein implicated in synaptic transmission. We also found that DYRK3 directly phosphorylates SNAPIN at the threonine (Thr) 14 residue, increasing the interaction of SNAPIN with other proteins such as dynein and synaptotagmin-1. In central nervous system neurons, SNAPIN is associated with and mediate the retrograde axonal transport of diverse cellular products from the distal axon terminal to the soma and the synaptic release of neurotransmitters, respectively. Moreover, phosphorylation of SNAPIN at Thr-14 was found to positively modulate mitochondrial retrograde transport in mouse cortical neurons and the recycling pool size of synaptic vesicles, contributing to neuronal viability. In conclusion, the present study demonstrates that DYRK3 phosphorylates SNAPIN, positively regulating the dynein-mediated retrograde transport of mitochondria and SNARE complex-mediated exocytosis of synaptic vesicles within the neurons. This finding further suggests that DYRK3 affects cell viability and provides a novel neuroprotective mechanism.

## Introduction

Dual-specificity tyrosine-(Y)-phosphorylation-regulated kinases (DYRKs), including DYRK1A, DYRK1B, DYRK2, DYRK3, and DYRK4, have similar structures and conserved catalytic domain sequence [1]; however, individual members have unique N-terminal DYRK-homology boxes and C-terminal domains responsible for substrate specificity and unique functions. DYRK1A is the most well-characterized member of the DYRK family to date, whereas the biochemical and functional properties of DYRK3 have not been studied in detail. A few substrates of DYRK3 were reported to have a role in cell protection. For example, upon activation, DYRK3 facilitates the dissolution of stress granules. DYRK3 directly phosphorylates the mTORC1 inhibitor PRAS40 at the threonine (Thr)-246 residue, releasing mTORC1 from the stress granules [2]. DYRK3 also increases cell survival by reducing p53 activity through the phosphorylation of SIRT1 at the Thr-522 [3]. Moreover, radiation-induced DYRK3 expression enhances mitochondrial fission via the activation of mTORC1-dependent DRP1 [4].

The synaptic release of neurotransmitters, including the sequential steps of vesicle docking and fusion, is mediated by the assembly of a stable synaptosomal-associated protein (SNAP) receptor (SNARE) core complex composed of at least 60 proteins such as synaptobrevin, syntaxin, SNAP25, and synaptotagmin. SNAPIN, a SNARE-associated protein, cooperates with the SNARE complexes to affect synaptic transmission. SNAPIN was first identified in neurons and is specifically located on the membranes of synaptic vesicles [5–7]. In *Snapin-*knockout mice, the connection between SNAP25 with synaptotagmin-1 was impaired, and calcium-dependent exocytosis was remarkably decreased [8]. Regarding the functional modulation of SNAPIN, protein kinase A (PKA) phosphorylates SNAPIN at serine (Ser)-50, enhancing its interaction with SNAP25 and the binding of synaptotagmin-1 to the SNARE complex [9]. In addition, familial Parkinson’s disease-linked LRRK2 phosphorylates SNAPIN at Thr-117, which inhibits the interaction between SNAPIN and SNAP25, causing a decrease in the number of readily releasable vesicles and the extent of exocytotic release in hippocampal neurons [10].

SNAPIN also acts as an adaptor that interacts with dyneins, a family of cytoskeletal motor proteins that move along microtubules for endosome trafficking to the lysosomes in neurons [11, 12]. In healthy neurons, autophagosomes are predominantly located in the distal axons. Through fusion with late endosomes to form autophagosomes, long-distance retrograde motility is achieved by recruiting the late endosome-loaded dynein-SNAPIN motor-adaptor transport machinery. This process is an essential mechanism in neurons that facilitates cargo degradation within autolysosomes in the soma, thus protecting against axonal stress. However, in the neurons of Alzheimer’s disease (AD) that are disturbed by amyloid β (Aβ) oligomers, SNAPIN reduces the interaction with dynein motors in the distal axon. As a result, late endosomes remain in the distal axons at the axon terminals, thereby increasing autophagic stress in AD neurons [13].

A recent report showed that DYRK3 is strongly expressed in many regions of the central nervous system (http://www.proteinatlas.org/ENSG00000143479-DYRK3); however, the additional substrates or binding regulators of DYRK3 and the underlying mechanism of its cytoprotective activity, especially within the neurons, remain to be elucidated. Indeed, there have only been a limited number of studies on DYRK3, mostly in actively proliferating cells. Therefore, the aim of the present study was to identify the novel binding protein(s) of DYRK3 through a yeast two-hybrid system. After screening the human brain cDNA library, we found that SNAPIN specifically binds to DYRK3, which directly phosphorylates SNAPIN at Thr-14. We further found that SNAPIN phosphorylation by DYRK3 stimulates SNAPIN-dynein-mediated retrograde transport activity, as well as SNARE complex-linked neurotransmitter release. The toxicity induced by serum deprivation in hippocampal progenitor H19-7 cells was mainly attributed to the loss of this novel and cytoprotective activity of DYRK3. These data further suggest that DYRK3 could be a potential therapeutic target for many SNAPIN-associated neuronal diseases.

## Materials and methods

### Cell culture and DNA transfection

Rat immortalized hippocampal progenitor H19-7 cells, human dopaminergic neuroblastoma SH-SY5Y cells, mouse embryonic fibroblasts (MEFs), and human embryonic kidney 293 (HEK293) cells were maintained in DMEM containing 10% FBS and 100 U/ml penicillin-streptomycin. Cells were grown at 37 °C in 5% CO_2_. All DNA transfections were performed using PEI and Lipofectamine 2000 reagents, according to the manufacturer’s protocol. The *Atg5*-knockout and control MEFs were kindly provided by Young J. Oh (Yonsei University, Seoul, Korea).

### Animals

Pregnant ICR mice and Sprague Dawley rats were purchased from Hyochang Science (Daegu-si, Republic of Korea) and Orient-Bio (Seongnam-si, Gyeonggi, Republic of Korea), respectively. All animal experiments were approved by the Institutional Animal Care and Use Committee (IACUC) of Pohang University of Science and Technology (POSTECH) and Seoul National University.

### Primary cortical neuron culture

Primary cultures of cortical neurons were established by isolating embryonic day 15 ICR mouse embryo cortical tissues in Hanks’ balanced salt solution (Gibco) and dissociating the tissues in 0.25% trypsin and 0.1% DNase I for 10 min at 37 °C. The cells were resuspended in neurobasal medium (Gibco) supplemented with 10 mM HEPES (pH 7.4) and 10% (v/v) horse serum to a final cell concentration of 3.0 × 10^5^ cells/ml, and then plated on glass coverslips pre-coated with poly-D-lysine and laminin (Corning). Two hours after plating, the cell medium was replaced with neurobasal medium containing 2 mM glutamine, 2% (v/v) B27 supplement (Gibco), and 1% (v/v) penicillin/streptomycin. Neurons were transfected with Lipofectamine 2000, and the medium was replaced with the culture medium 4 h after transfection.

### Mitochondrial transport imaging and analysis

Mitochondrial transport imaging was performed as previously described, with modifications [14, 15]. Primary cultured cortical neurons at day in vitro (DIV) 7 were transfected with the Mito-GFP construct, as indicated. At DIV 9-11, live time-lapse imaging was performed using an FV3000 confocal laser scanning microscope (Olympus) with an UPLSAPO 20×/0.75 NA objective at 37 °C supplied with 5% CO_2_ gas. Neurons were imaged for 2 min with 3-sec intervals. The acquired images were subjected to mitochondrial motility analysis. The axon was identified as a long and thin process and a 150 µm axonal segment from at least 100 μm away from the soma was selected and analyzed. Mitochondria showing displacement from the original point of at least 5 μm for 2 min were regarded as motile. Motile and stationary mitochondria were counted manually based on image sequences and kymographs generated using Cell Sens (Olympus). Mitochondrial motility is presented as the percentage of motile mitochondria to total mitochondria. The velocity of moving mitochondria was analyzed using ImageJ using the KymoAnalyzer v1.01 plug-in [16].

### Statistical analyses

The S-Group means of samples were compared using Student’s t-test or one-way analysis of variance (ANOVA), and *p* < 0.05 was considered statistically significant. Values are reported as mean ± standard error of the mean (SEM) or standard deviation (SD). The densities of the western blot bands were measured using GelQuant.NET software (version 1.8.2), according to the protocol provided by the manufacturer (http://www.biochemlabsolutions.com).

*More information on the Materials and Methods is available in the Supplementary Information*.

## Results

### Serum deprivation rapidly reduced intracellular DYRK3 level in HEK293 and H19-7 cells

Based on the previous report demonstrating that DYRK3 regulates mTORC1 signaling through the phosphorylation of PRAS40 under stressful condition, thereby affecting cell growth and viability [2], we hypothesized that DYRK3 exerts cytoprotective activity. To further verify this hypothesis, we examined whether intracellular DYRK3 levels could be altered in response to various stress inducers. When HEK293 cells were treated with one of these drugs, intracellular DYRK3 level was not notably affected, except under the condition of serum deprivation, which caused the complete loss of DYRK3 expression (Supplementary Fig. S1A, B). Next, we examined whether serum deprivation decreased DYRK3 levels in mammalian neuronal cells, using H19-7 cells as the model. As shown in Supplementary Fig. S1C, D, serum deprivation of H19-7 cells also caused the complete loss of DYRK3 expression. Moreover, serum deprivation in HEK293 cells resulted in the rapid loss of DYRK3 at 1 min, which was completely eliminated by 30 min (Supplementary Fig. S1E, F). Interestingly, after cells were exposed to serum deprivation for 2 h, followed by a change of medium including 10% FBS, the DYRK3 level fully recovered to the level of control cells without any treatment (Supplementary Fig. S1G, H). In addition, the recovery of completely reduced DYRK3 level by serum deprivation occurred in a dose-dependent manner with respect to the FBS supplemented within the medium (Supplementary Fig. S1I, J).

Next, we assessed the subcellular localization of DYRK3 and the effect of serum deprivation on these changes. After serum deprivation, HEK293 cell lysates were separated into cytosolic and nuclear fractions, and DYRK3 was equally expressed in the cytosolic and nuclear regions. However, cytosolic DYRK3 disappeared under serum deprivation and was restored to the control level by the addition of FBS. This effect was not observed in the nuclear fraction of DYRK3 (Supplementary Fig. S1K, L). Taken together, these results indicated that serum deprivation causes a rapid reduction in cytosolic DYRK3 levels in mammalian cells.

### Serum deprivation triggers an autophagic response for DYRK3 degradation as well as a decrease in the *Dyrk3* mRNA level

Next, we attempted to identify the mechanism by which serum deprivation reduced DYRK3 levels. The ubiquitin-proteasome system (UPS) and autophagy-lysosomal pathway are two major degradative pathways of proteins in eukaryotic cells, which play critical roles in cell survival under normal conditions, as well as in response to a variety of stresses. Therefore, we tested whether serum deprivation activates either one or both proteolysis systems, and if that is the case, which pathway is applied to DYRK3 degradation, using a specific inhibitor of proteasome and autophagy, respectively. If these two pathways are not involved in the degradation of DYRK3, the activation of apoptotic death-inducing caspases could be accompanied by serum deprivation. Therefore, we examined the effect of pretreatment with proteasome, autophagy or pan-caspase inhibitors on the serum-deprivation-induced reduction of DYRK3. As shown in Supplementary Fig. S2A, B, the reduction in DYRK3 levels by serum deprivation was not rescued by treatment with the proteasome inhibitor MG132, indicating that serum deprivation does not activate the proteasome machinery. For this experiment, cells were treated with 5% FBS-containing DMEM instead of subjected to the condition of complete serum deprivation because DMEM treatment caused a rapid and complete reduction of DYRK3, and the cells were not considerably influenced by the drug. When the cells were pretreated with Z-VAD, the substantially reduced DYRK3 levels did not recover (Supplementary Fig. S2C, D). Lastly, pretreatment with the autophagy inhibitor NH_4_Cl restored the DYRK3 level reduced by low serum treatment, indicating that serum reduction or starvation causes DYRK3 degradation via activating the autophagy pathway (Supplementary Fig. S2E, F). We also found that the reduction in the endogenous DYRK3 level was restored to the control level in a dose-dependent manner with respect to NH_4_Cl (Supplementary Fig. S2G, H).

Autophagy consists of several sequential steps, including the formation of autophagosomes that mediate the delivery of cytoplasmic cargo to the lysosomes. Autophagosome formation is dynamically regulated by multiple autophagy-related (ATG) protein complexes, including ATG5 [17]. In *Atg5*-knockout cells, autophagy progression does not occur. Therefore, to verify the involvement of the autophagy pathway in the proteolysis of DYRK3, we examined whether DYRK3 degradation could occur under serum deprivation in *Atg5*-knockout MEFs. As shown in Supplementary Fig. S2I, J, DYRK3 was completely degraded under serum deprivation in wild-type MEFs as a control. However, the degradation of DYRK3 was significantly diminished in *Atg5*-knockout cells compared with that of wild-type MEFs (Supplementary Fig. S2J). Since the DYRK3 level in *Atg5*-knockout cells was not fully restored to the control level, we further checked whether serum starvation alters the gene expression of *Dyrk3*. Real-time PCR revealed that the *Dyrk3* mRNA levels rapidly decreased under serum deprivation for 30 min and were maintained for 1 h (Supplementary Fig. S2K).

These data suggested that in serum-deprived cells, DYRK3 levels rapidly decrease via activation of the autophagy pathway to mediate DYRK3 degradation as well as via the reduction of *DYKR3* gene transcription.

### DYRK3 interacts with SNAPIN in mammalian cells

**Fig. 1:**
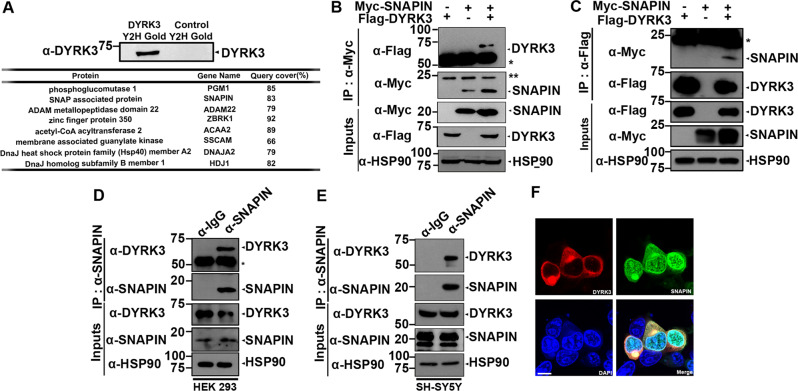
DYRK3 interacts with SNAPIN in mammalian cells. **A** Yeast two hybrid assays were performed using Matchmaker Gold System and the Y2HGold yeast strain co-transformed with pGBKT7-DYRK3 plasmid and pGBKT7-prery vector. The expression of the bait vector was confirmed by immunoblotting with anti-DYRK3 antibody. After screening the human brain library, positive clones were selected by growth on the media deficient of selective amino acids. The binding-candidate cDNAs from prey vectors were verified by PCR amplification using the T7 sequencing primer. The table below denotes the protein names and gene symbols of screened products. **B** HEK293 cells were transfected for 24 h with a plasmid encoding Myc-SNAPIN or Flag-DYRK3 alone or in combination. Cell lysates were immunoprecipitated with anti-Myc antibody, followed by immunoblotting with the indicated antibody. Hsp90 served as a loading control. The asterisk indicates IgG heavy and light chain. **C** HEK293 cells were transfected for 24 h with a plasmid encoding Myc-SNAPIN or Flag-DYRK3 alone or in combination. Cell lysates were immunoprecipitated with anti-Flag antibody, followed by immunoblotting with the indicated antibody. The asterisk indicates IgG light chain. **D**, **E** HEK293 **D** and SH-SY5Y cell lysates **E** were immunoprecipitated with either anti-SNAPIN antibody or pre-immune IgG as a control, followed by immunoblotting with the indicated antibody. The asterisk indicates IgG heavy chain. **F** Representative confocal images of immunostaining of HEK293 cells expressing both Flag-DYRK3 (red) and GFP-SNAPIN (green). Nuclei were counterstained with DAPI (blue). Scale bar = 10 μm.

To identify the molecular mechanism underlying the cytoprotective effect of DYRK3 and its functional regulation, novel DYRK3-interacting proteins were screened using a yeast two-hybrid assay employing DYRK3 as bait (Fig. 1A). After screening the human brain library, several DYRK3 binding partners in yeast were identified, including phosphoglucomutase 1 (PGM1), ADAM22, and SNAPIN. Moreover, zinc finger protein 350, acetyl CoA acyltransferase, membrane-associated guanylate kinase, and two members of the DnaJ heat-shock protein family were found to be novel binding proteins of DYRK3. Although PGM1 plays a fundamental role in glycolysis, glycogenesis, and glycogenolysis [18], both ADAM22 and SNAPIN are closely related to the synaptic transmission of neurotransmitters [19]. Among these proteins, SNAPIN was chosen as a target of further analysis because some reports demonstrated that proper action of SNAPIN is critical for neuronal homeostasis, which is primarily attributed to the efficient axonal retrograde transport of various molecules via the mitochondria and autophagosome, as well as by precise synaptic neurotransmitter secretion [9, 20].

Therefore, we investigated whether and how SNAPIN is biochemically and functionally linked to DYRK3. Co-immunoprecipitation (Co-IP) analysis revealed that ectopically expressed DYRK3 binds to SNAPIN (Fig. 1B). When the Co-IP assay was performed in the reverse order, we observed the same result (Fig. 1C). Next, Co-IP analyses using anti-SNAPIN and anti-DYRK3 antibody revealed that endogenous DYRK3 was associated with endogenous SNAPIN in SH-SY5Y and HEK293 cells (Fig. 1D, E). These results suggested that the DYRK3-SNAPIN interaction is not an artifact of DNA transfection but is rather a specific interaction in mammalian cells. Moreover, immunostaining of cells with anti-Flag (red) and anti-GFP (green) antibodies revealed that both proteins are commonly expressed in the cytosol (Fig. 1F). These data suggest that DYRK3 directly interacts with SNAPIN in mammalian cells.

### DYRK3 phosphorylates SNAPIN at the Thr-14 residue

**Fig. 2:**
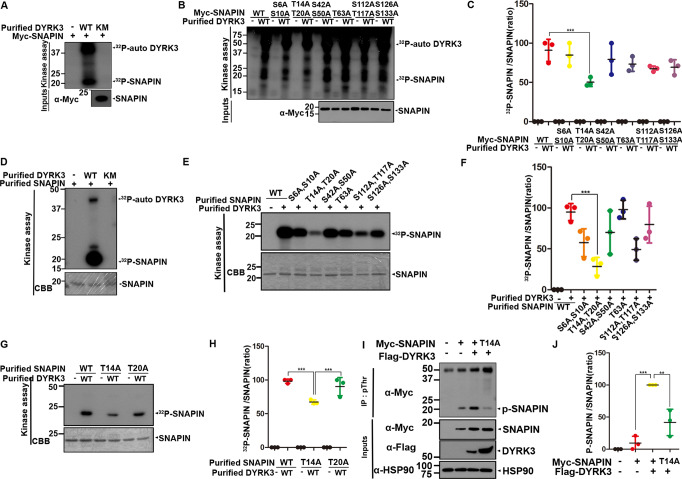
DYRK3 phosphorylates SNAPIN at the threonine 14 residue. **A** After HEK293 cells were transfected for 24 h with a plasmid encoding Myc-SNAPIN, three equal cell lysates (~1000 μg of protein) were immunoprecipitated with anti-Myc antibody. Where specified, the samples were mixed with bacterially expressed wild-type DYRK3 (DYRK3-WT) or its kinase-inactive mutant (DYRK3-KM), incubated for 30 min at 30 °C with kinase buffer and [γ-^32^P]ATP, resolved by SDS-PAGE, and analyzed by autoradiography. Proper expression of transiently expressed SNAPIN in cell extracts was verified by immunoblotting with anti-Myc antibody (Input). **B** HEK293 cells were transfected for 24 h with a plasmid encoding Myc-SNAPIN-WT or one of the SNAPIN point-mutants, including SNAPIN-S6/S10A, SNAPIN-T14/T20A, SNAPIN-S42/S50A, SNAPIN-T63A, SNAPIN-S112/T117A, and SNAPIN-S126/S133A, followed by immunoprecipitation with anti-Myc antibody. The anti-Myc immunocomplexes as a substrate were mixed with bacterially expressed DYRK3-WT, incubated for 30 min at 30 °C with kinase buffer and [γ-^32^P]ATP, resolved by SDS-PAGE, and analyzed by autoradiography. **C** Quantification of expression levels from the blots in **B**. Data represent the mean ± standard deviation of three independent experiments (***p < 0.001). Statistical analyses were performed using the IBM SPSS Statistics software (version 23.0). **D**, **E** In vitro kinase assays mixing the bacterially expressed DYRK3-WT, DYRK3-KM, SNAPIN-WT **D**, and one of several SNAPIN-point mutants **E** alone or in combination with kinase buffer and [γ-^32^P]ATP, incubated for 30 min at 30 °C, resolved by SDS-PAGE, and analyzed by autoradiography. The recombinant SNAPIN protein bands were visualized with Coomassie blue staining (CBB). **F** Quantification of the bands in **E**. All data represent the mean ± standard deviation of three independent experiments (***p < 0.001). **G** In vitro kinase reaction products were prepared by mixing bacterially expressed DYRK3-WT, SNAPIN-T14A, or SNAPIN-T20A alone or in combination, incubated for 30 min at 30 °C with kinase buffer and [γ-^32^P]ATP, resolved by SDS-PAGE, and analyzed by autoradiography. The recombinant SNAPIN bands were visualized with CBB. **H** Quantification of the bands in **G**. All data represent the mean ± standard deviation of three independent experiments (***p < 0.001). **I** HEK293 cells were transfected for 24 h with a plasmid encoding Myc-SNAPIN WT, Myc-SNAPIN-T14A, or Flag-DYRK3 alone or in combination. Cell lysates were immunoprecipitated with anti-pThr antibody, followed by immunoblotting with anti-Myc antibody. **J** Quantification of the bands in **I**. All data represent the mean ± standard deviation of three independent experiments (**p < 0.01, ***p < 0.001).

To gain further insight into the link between DYRK3 and SNAPIN, we attempted to determine whether DYRK3 directly phosphorylates SNAPIN. To this end, we employed an in vitro kinase assay (Fig. 2A–F). As shown Fig. 2A, wild-type DYRK3 phosphorylated SNAPIN, whereas phosphorylation was not observed with its kinase-inactive mutant with the K238M substitution (DYRK3-KM).

Next, we attempted to identify the exact DYRK3-targeting site(s) in SNAPIN. Dual-specificity protein kinases, including DYRK3, undergo tyrosine auto-phosphorylation and catalyze the phosphorylation of substrates on serine/threonine residues. As SNAPIN contains a total of 11 serine (S) and threonine (T) residues, six point-mutants of SNAPIN with single or double mutations of these sites with alanine (Ala, A) were generated. We then carried out an in vitro kinase assay using the anti-Myc-SNAPIN immunocomplexes as a substrate alone or together with bacterial recombinant DYRK3-WT as a kinase, measured the phosphorylation status of SNAPIN, and compared the phosphorylation level of each mutant with that of wild-type SNAPIN as a control (Fig. 2B, C). Among six mutants, the SNAPIN-T14/T20A mutant displayed the most reduced phosphorylation level, which was ~50% lower than that of wild-type SNAPIN.

To verify the occurrence of phosphorylation of these six SNAPIN mutants by DYRK3, we performed in vitro kinase assays using each bacterially recombinant mutant protein as a substrate. Autoradiographic analyses of the reaction products showed the same pattern as found previously, in which DYRK3-WT, but not DYRK3-KM, directly phosphorylated SNAPIN (Fig. 2D). In addition, among the six recombinant SNAPIN mutants, the phosphorylation level of the SNAPIN-T14/T20A mutant was significantly reduced compared with that of wild-type SNAPIN (Fig. 2E, F). However, the extent of protein phosphorylation in the other five mutants did not significantly change.

To determine whether a single site between T14 and T20 could be preferentially phosphorylated, we created two mutants with a single mutation of T14 and T20. In vitro kinase assays revealed that SNAPIN-T14A displayed the most reduced phosphorylation signal compared with those of SNAPIN-WT and SNAPIN-T20A (Fig. 2G, H). We then determined whether this occurs within the cells. Co-IP assays demonstrated that the amount of phosphorylation at Thr by DYRK3 was significantly increased in SNAPIN-WT, but not in the SNAPIN-T14A mutant (Fig. 2I, J).

Taken together, these data indicated that DYRK3 directly phosphorylates SNAPIN at Thr-14 residue.

### DYRK3-mediated phosphorylation of SNAPIN increases its binding to dynein

**Fig. 3:**
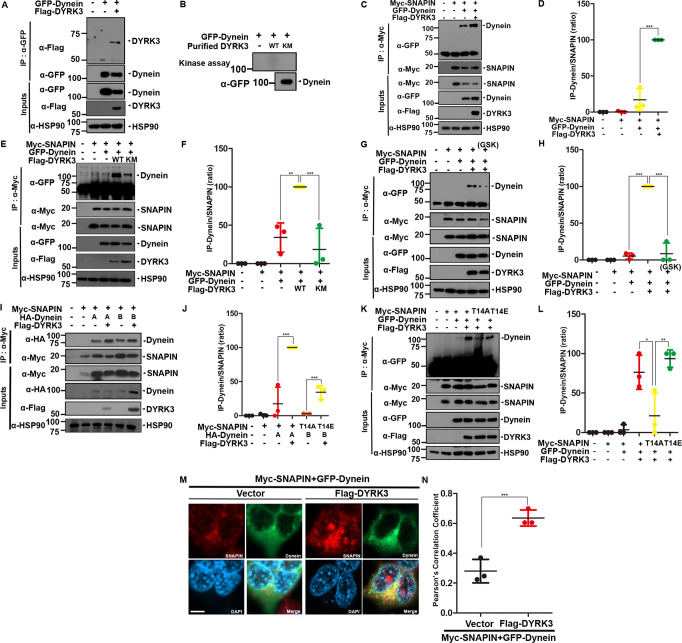
DYRK3-mediated phosphorylation of SNAPIN at Thr-14 increase the binding of SNAPIN to dynein. **A** After HEK293 cells were transfected for 24 h with a plasmid encoding GFP-Dynein or/and Flag-DYRK3, cell lysates were immunoprecipitated with anti-GFP antibody, followed by immunoblotting with the indicated antibody. Hsp90 served as a loading control. **(B)** After HEK293 cells were transfected for 24 h with a plasmid encoding GFP-Dynein, cell lysates (~1,000 μg of protein) were immunoprecipitated with anti-GFP antibody. For the in vitro kinase assay, the anti-GFP immunocomplex as a substrate was mixed with bacterially expressed DYRK3-WT or DYRK3-KM, and incubated for 30 min at 30^o^C with kinase buffer and [γ-^32^P]ATP, resolved by SDS-PAGE, and analyzed by autoradiography. Proper expression of transiently expressed dynein in cell extracts was verified by western blotting with anti-GFP antibody (Input). **C** HEK293 cells were transfected for 24 h with a plasmid encoding Myc-SNAPIN, GFP-Dynein, or Flag-DYRK3 alone or in combination. Cell lysates were immunoprecipitated with anti-Myc antibody, followed by immunoblotting with the indicated antibody. **D** Quantification of the blots in **C**. Data represent the mean ± standard deviation of three independent experiments (***p < 0.001). **E** Where specified, HEK293 cells were transfected for 24 h with a plasmid encoding Myc-SNAPIN, GFP-Dynein, Flag-DYRK3-WT, or Flag- DYRK3-KM alone or in combination. Cell lysates were immunoprecipitated with anti-Myc antibody, followed by immunoblotting with the indicated antibody. **F** Quantification of the blots in **E**. All data represent the mean ± standard deviation of three independent experiments (**p < 0.01, ***p < 0.001). **G** After HEK293 cells were transfected for 24 h with a plasmid encoding Myc-SNAPIN, GFP-Dynein, or Flag-DYRK3-WT alone or in combination, the cells were left untreated or treated for 6 h with 1 μM of GSK-626616, a DYRK3 kinase-specific chemical inhibitor. Cell lysates were immunoprecipitated with anti-Myc antibody, followed by immunoblotting with the indicated antibody. **H** Quantification of the blots in **G**. All data represent the mean ± standard deviation of three independent experiments (***p < 0.001). **I** HEK293 cells were transfected for 24 h with a plasmid encoding Myc-SNAPIN, HA-Dynein A, HA-Dynein B, or Flag-DYRK3-WT alone or in combination. Cell lysates were immunoprecipitated with anti-Myc antibody, followed by immunoblotting with the indicated antibody. **J** Quantification of the blots in **I**. All data represent the mean ± standard deviation of three independent experiments (***p < 0.001). **K** After HEK293 cells were transfected for 24 h with a plasmid encoding Myc-SNAPIN, Myc-SNAPIN-T14A, Myc-DNAPIN-T14E, GFP-Dynein, or Flag-DYRK3-WT alone or in combination, cell lysates were immunoprecipitated with anti-Myc antibody, followed by immunoblotting with the indicated antibody. **L** Quantification of the blots in **K**. All data represent the mean ± standard deviation of three independent experiments (*p < 0.05, **p < 0.01). **M** HEK293 cells were transfected for 24 h with a plasmid encoding Flag-DYRK3. Representative confocal images of immunostaining of HEK293 cells expressing both Myc-SNAPIN (red) and GFP-Dynein (green). Nuclei were counterstained with DAPI (blue). Scale bar = 10 μm. **N** Pearson’s correlation coefficient for assessing the co-localization of SNAPIN and dynein was calculated using Image J software. All data represent the mean ± standard deviation of three independent experiments (***p < 0.001).

Dynein is the primary motor protein that drives the retrograde transport of organelles, endosomes, and autophagic vacuoles from distal axons to the soma along the microtubules. SNAPIN serves as an adaptor for the recruitment of dynein motors to the endosomes, and disruption of dynein-SNAPIN coupling impairs the retrograde axonal transport of endosomes [21]. We investigated whether the phosphorylation of SNAPIN at Thr-14 by DYRK3 affects the interaction between SNAPIN and dynein. We first tested whether DYRK3 also binds to and phosphorylates dynein as a control. Co-IP analyses revealed that ectopically expressed dynein binds to DYRK3 (Fig. 3A). The in vitro kinase assay revealed that DYRK3 did not phosphorylate dynein (Fig. 3B). These results indicated that DYRK3 binds to dynein, but does not directly phosphorylate it.

Next, we examined whether DYRK3 affects the interaction of SNAPIN with dynein. Co-IP analyses revealed that Flag-SNAPIN binds to GFP-dynein in the resting state, as expected. In addition, the interaction between SNAPIN and dynein was significantly increased in the presence of wild-type DYRK3 (Fig. 3C, D), but not in the presence of DYRK3-KM (Fig. 3E, F). Moreover, when cells were treated with the DYRK3-specific chemical inhibitor GSK626616, the binding between SNAPIN and dynein was similarly decreased (Fig. 3G, H). These data suggest that the kinase activity of DYRK3 contributes to enhancing the binding affinity between SNAPIN and dynein.

The dynein can be divided into two groups: cytoplasmic and axonal one. In addition to GFP-dynein-A and HA-dynein-A, we found that the binding of another cytoplasmic dynein isoform, HA-dynein-B, to SNAPIN was similarly enhanced when cells were co-transfected with Flag-DYRK3-WT (Fig. 3I, J). We next examined whether the phosphorylation of SNAPIN at Thr-14 influences the binding affinity of SNAPIN to dynein. Co-IP analyses revealed that The T14-phosphorylation-defective SNAPIN mutant (SNAPIN-T14A) displayed substantially reduced binding to dynein than SNAPIN-WT (Fig. 3K, L). In contrast, the phospho-mimetic SNAPIN-T14E mutant exerted enhanced binding to dynein, showing a similar level as that found for SNAPIN-WT, compared with that of SNAPIN-T14A. Confocal microscopic analysis additionally revealed that control cells in the absence of DYRK3 displayed no significant interaction between SNAPIN and dynein. However, there was an increase in the binding of SNAPIN to dynein in cells overexpressing Flag-DYRK3 (Fig. 3M, N).

Taken together, these results suggested that the phosphorylation of SNAPIN at Thr-14 by DYRK3 enhances its binding affinity with dynein.

### Phosphorylation of SNAPIN at Thr-14 regulates mitochondrial retrograde transport in mouse cortical neurons

**Fig. 4:**
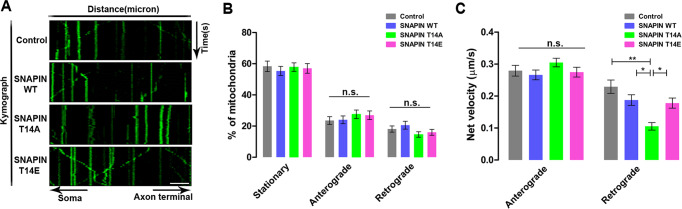
Phosphorylation of SNAPIN at Thr-14 regulates mitochondrial retrograde transport in mouse cortical neurons. **A** Representative kymographs of mitochondrial movement in mouse primary cortical neuron transfected with SNAPIN-WT, SNAPIN-T14A, or SNAPIN-T14E. The movement of mitochondria is shown. Scale bar = 10 μm. **B** Average motility of mitochondria. Motility is presented as the percentage of motile mitochondria over total mitochondria. Anterograde and retrograde motility were assessed, respectively (*n* = 29 axons for control [CTL], *n* = 33 axons for SNAPIN wild-type [WT], *n* = 34 axons for SNAPIN-T14A mutant, and *n* = 31 axons for SNAPIN-T14E mutant). All results are presented as the mean ± SEM. (n.s., not significant) analyzed with Student’s t test. **C** Average velocity of motile mitochondria. Anterograde and retrograde velocity were assessed separately. All results are presented as the mean ± SEM. *p < 0.05, **p < 0.01 and from Student’s t test.

According to a previous report, axonal retrograde transport involves a motor protein complex comprising cytoplasmic dynein that carries out the retrograde transport of mitochondria toward the soma of neurons [22, 23]. As SNAPIN has also been reported to regulate axonal mitochondrial movement, we explored whether phosphorylation of SNAPIN at Thr-14 affects axonal mitochondrial movement in cooperation with the dynein complex. Therefore, we analyzed the role of SNAPIN-T14 phosphorylation in the axonal transport of the mitochondria in primary cultured cortical neurons from mouse embryos. When compared to that induced by wild-type SNAPIN using mitochondrial kymographs (Fig. 4A), the mean velocity of retrograde movement was significantly impaired by overexpression of the phospho-deficient SNAPIN-T14A mutant without affecting stationary and motile fractions of mitochondria, whereas the anterograde velocity remained intact (Fig. 4B, C). Mitochondrial movement appeared to be more sensitive to the loss of SNAPIN phosphorylation, as this defect was not observed in the presence of the phospho-mimetic SNAPIN-T14E mutant.

These results further support the notion that the dynein-dependent mitochondrial movement in the axon is regulated by SNAPIN Thr-14 phosphorylation. In addition, phosphorylation of SNAPIN at Thr-14 by DYRK3 appears to be critical for axonal retrograde transport in cells.

### The phosphorylation of SNAPIN at Thr-14 by DYRK3 increases its binding to synaptotagmin-1 in the SNARE complex

In addition to dynein, SNAPIN was initially reported to be a component of the multiprotein SNARE complex assembled at the presynaptic terminal, including syntaxin, complexin, SNAP25, and Ca^2+^ sensor protein [5]. Interestingly, the phosphorylation of SNAPIN at Thr-117 by LRRK2 affects the interaction of SNAPIN with SNAP25 and the extent of exocytotic neurotransmitter release. Thus, we hypothesized a regulatory role of DYRK3 in the presynaptic transmission of neurotransmitter vesicles through the modification of the SNAPIN-SNARE complex. To verify this hypothesis, we further examined whether phosphorylation SNAPIN at Thr-14 by DYRK3 regulates exocytotic release via its interaction with SNAP25. Similar to the experiment with dynein, before evaluating the relationship of SNAPIN with SNAP25 mediated by DYRK3, we first determined whether DYRK3 directly regulates SNAP25. Immunoblot analysis revealed that SNAP25 binds to DYRK3 (Supplementary Fig. S3A). The in vitro kinase assay revealed that DYRK3 did not directly phosphorylate SNAP25 (Supplementary Fig. S3B). These results indicate that DYRK3 interacts with SNAP25 but does not phosphorylate SNAP25 directly, suggesting that the function of SNAP25 is not affected by DYRK3.

Next, we examined whether DYRK3 affects the interaction of SNAPIN with SNAP25. Co-IP revealed that the binding of SNAPIN to SNAP25 did not change significantly, irrespective of using DYRK3-WT or DYRK3-KM (Supplementary Fig. S3C, D). We next examined whether phosphorylation at Thr-14 influences the binding affinity of SNAPIN to SNAP25. Co-IP analyses revealed that the binding of SNAP25 to SNAPIN-T14A or SNAPIN-T14E mutant did not change at all compared with that of SNAP-WT (Supplementary Fig. S3E, F). These data suggest that the phosphorylation of SNAPIN at Thr-14 does not affect its interaction with SNAP25.

In addition to SNAP25, we further examined whether SNAPIN phosphorylation affects its interaction with other components of SNARE protein complexes, such as synaptotagmin-1, a Ca^2+^ sensor in the membrane of the pre-synaptic axon terminal (Supplementary Fig. S3G, H). In vitro pull-down assays revealed that the binding of the SNAPIN-T14A mutant to synaptotagmin-1 was greatly reduced compared with that of SNAPIN-WT or the SNAPIN-T14E mutant. These results indicated that phosphorylation of SNAPIN at Thr-14 increases the binding of SNAPIN to synaptotagmin-1 in the SNARE complex.

### DYRK3-mediated phosphorylation of SNAPIN positively modulates the neurotransmitter release

**Fig. 5:**
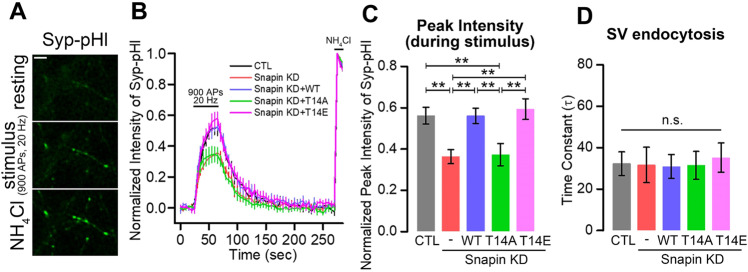
DYRK3-mediated phosphorylation of SNAPIN at Thr-14 appears to positively modulate neurotransmitter release. **A** Representative images of cultured rat hippocampal neurons transfected with Syp-pHl at day in vitro (DIV) 14 during the resting state, under stimulation (900 action potentials [APs] at 20 Hz), and the application of 50 mM NH_4_Cl to de-quench the total synaptic vesicle pool. Scale bar = 5 μm. **B** Average normalized fluorescent intensity of Syp-pHl during the resting state, under stimulation (900 APs at 20 Hz), and with application of 50 mM NH_4_Cl in control (CTL), *SNAPIN*-knockdown (Snapin-KD), Snapin-KD + Snapin-wild type (Snapin-KD + WT), Snapin-KD + Snapin-T14A (Snapin-KD + T14A), and Snapin-KD + Snapin-T14E (Snapin-KD + 14E) neurons. Values are indicated as mean ± SEM. **C** Average normalized peak intensity of Syp-pHl during stimulation in CTL, Snapin-KD, Snapin-KD + WT, Snapin-KD + T14A, and Snapin-KD + T14E neurons. CTL, 0.561 ± 0.041, *n* = 6 (coverslips); Snapin-KD, 0.363 ± 0.035, *n* = 5 (coverslips); Snapin-KD + WT, 0.561 ± 0.037, *n* = 5 (coverslips); Snapin-KD + T14A, 0.373 ± 0.054, n = 5 (coverslips); Snapin-KD + T14E, 0.593 ± 0.050, *n* = 5 (coverslips); F(4, 21) = 6.462, *p* = 0.001, one-way ANOVA, followed by a least significant difference (LSD) post-hoc test. **D** Average time constant (τ) of Syp-pHl after stimulation in CTL, Snapin-KD, Snapin-KD + WT, Snapin-KD + T14A, and Snapin-KD + T14E neurons. CTL, 32.287 ± 5.692 sec, *n* = 6 (coverslips); Snapin-KD, 31.664 ± 8.528 sec, *n* = 5 (coverslips); Snapin-KD + WT, 30.891 ± 5.790 sec, *n* = 5 (coverslips); Snapin-KD + T14A, 31.400 ± 6.748 sec, *n* = 5 (coverslips); Snapin-KD + T14E, 35.180 ± 7.079 sec, *n* = 5 (coverslips); F (4, 21) = 0.060, *p* = 0.993, one-way ANOVA followed by an LSD post-hoc test. Values are indicated as mean ± SEM; **p < 0.01. n.s., not significant.

To address the physiological effect of SNAPIN phosphorylation on synaptic vesicle recycling, cultured rat hippocampal neurons were co-transfected with siRNA targeting *SNAPIN* or control siRNA along with synaptophysin1-pHluorin (Syp-pHl), a reliable pH-sensitive fluorescent sensor whose fluorescence is quenched in the acidic vesicular lumen but increases upon extracellular exposure [24] (Fig. 5A). When the siRNA-transfected neurons were stimulated with 900 APs at 20 Hz to completely deplete the recycling pool, the fluorescence intensity of Syp-pHl increased rapidly and decayed after stimulation, representing synaptic vesicle exocytosis and endocytosis, respectively (Fig. 5B). The heterogeneity of the total pool size among different boutons was compensated by normalization to the signal obtained after treatment with NH_4_Cl, which reveals the total vesicle pool size of the given boutons. The peak intensity of Syp-pHl in the sample with *SNAPIN-*knockdown (*Snapin*-KD) during stimulation was significantly smaller than that in the control neurons, indicating that the recycling pool size was reduced with *Snapin*-KD. However, the endocytic kinetics, as monitored by an exponential decay constant, remained unaltered in *Snapin*-KD neurons compared with those of control neurons, which is consistent with a previous report [25]. We further found that introducing SNAPIN-WT or SNAPIN-T14E mutant in the background of *Snapin*-KD fully restored the recycling pool size in neurons with *Snapin*-KD, while SNAPIN-T14A failed to do so, indicating that phosphorylation at Thr-14 is important for SNAPIN to regulate the recycling pool size of synaptic vesicle (Fig. 5C, D).

Collectively, our results indicate that phosphorylation of Thr-14 increases the binding of SNAPIN to synaptotagmin-1 in the SNARE complex, thereby increasing synaptic neurotransmitter release. DYRK3 also appears to play an important role in the modulation of synaptic transmission in neurons though SNAPIN phosphorylation.

### Serum deprivation-induced decrease in DYRK3 and sequential SNAPIN phosphorylation contribute cell toxicity

**Fig. 6:**
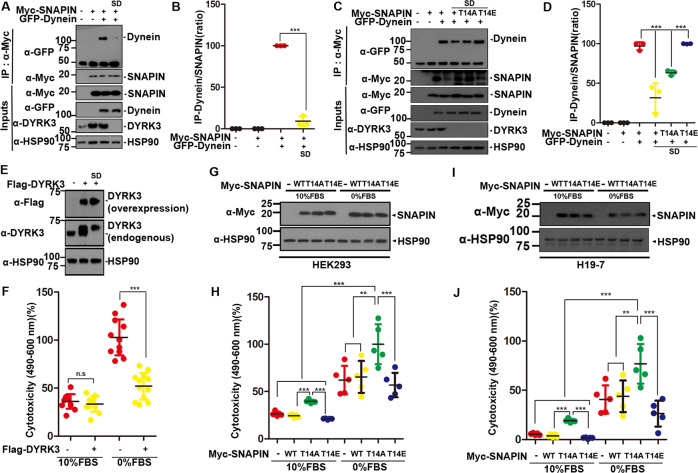
Blockade of SNAPIN phosphorylation via the reduction of DYRK3 contributes to cell toxicity under serum deprivation. **A** After HEK293 cells were transfected for 24 h with a plasmid encoding Myc-SNAPIN or GFP-Dynein alone or in combination, the cells were left untreated or treated for additional 6 h with DMEM. Cell lysates were immunoprecipitated with anti-Myc antibody, followed by immunoblotting with the indicated antibody**. B** Quantification of the blots in (A). All data represent the mean ± standard deviation of three independent experiments (***p < 0.001). **C** HEK293 cells were transfected for 24 h with a plasmid encoding Myc-SNAPIN, Myc-SNAPIN-T14A, Myc-DNAPIN-T14E, or GFP-Dynein alone or in combination, and were left untreated or treated for 6 h with DMEM. Cell lysates were immunoprecipitated with anti-Myc antibody, followed by immunoblotting with the indicated GFP antibody. **D** Quantification of the blots in **C**. All data represent the mean ± standard deviation of three independent experiments (***p < 0.001). **E** HEK293 cells were mock-transfected (-) or transfected for 24 h with a plasmid encoding Flag-DYRK3 alone, and cells were immunoblotted using anti-Flag or anti- DYRK3 antibody. Hsp90 served as a loading control. **G**, **I** After HEK293 **G** or H19-7 cells **I** were transfected for 24 h with a plasmid encoding Myc-SNAPIN, Myc-SNAPIN-T14A, or Myc-SNAPIN-T14E, the cells were left untreated or treated for an additional 6 h with DMEM. Cells were immunoblotted using the indicated antibody. **F, H, J** Where specified, the cytotoxicity from the samples in **E**, **G**, and **I** was measured by the amount of LDH released into the cultured media, respectively. The graph represents the mean ± standard deviation of twelve-independent experiments (**p < 0.01, ***p < 0.001, n.s., not significant).

Finally, we examined whether serum deprivation affects the interaction between SNAPIN and dynein after SNAPIN phosphorylation by DYRK3. Co-IP analyses revealed that serum deprivation caused a reduction in the interaction of SNAPIN with dynein (Fig. 6A, B). This effect was shown to occur through the reduction of endogenous DYRK3 levels (Fig. 6A), consequently decreasing SNAPIN phosphorylation under serum starvation. Eventually, non-phosphorylated SNAPIN would have weaker binding affinity for dynein. Next, we examined whether phosphorylation at Thr-14 could influence the binding affinity of SNAPIN to dynein under serum deprivation. Co-IP assays revealed that cells with the SNAPIN-T14E mutant displayed improved binding affinity of SNAPIN to dynein, similar to the level of cells with SNAPIN-WT observed under normal culture conditions (Fig. 6C, D). However, this effect was not observed in cells treated with SNAPIN-T14A under the serum-deprived condition. These results indicated that serum deprivation decreases the binding of SNAPIN to dynein by blocking SNAPIN phosphorylation at Thr-14.

We next examined whether the steady-state level of DYRK3 and/or SNAPIN phosphorylation by DYRK3 is critical for cell survival. Unlike endogenous DYRK3, ectopically expressed DYRK3 was unaffected by serum deprivation (Fig. 6E). The LDH assay confirmed that cells overexpressing DYRK3 showed much greater protection against serum deprivation-induced cytotoxicity than control cells (Fig. 6F). In addition, we examined whether SNAPIN-phosphorylation at Thr-14 affects serum deprivation-induced cytotoxicity in HEK293 and rat hippocampal H19-7 cells (Fig. 6G, I). The presence of SNAPIN-T14A caused considerably enhanced cytotoxicity in HEK293 and H19-7 cells under serum deprivation. (Fig. 6H, J). However, both cell lines with SNAPIN-WT and SNAPIN-T14E mutant showed substantially reduced cytotoxicity under serum deprivation.

Taken together, these data demonstrate that the DYRK3-mediated SNAPIN phosphorylation and subsequent increase in axonal retrograde transport contribute to the reduction in cellular toxicity under serum deprivation.

## Discussion

Among the five members of the DYRK family, which are involved in the regulation of cell migration, proliferation, and differentiation [26, 27], DYRK3 plays a key role in cell survival [3, 28]. DYRK3 is also activated when cells are exposed to various stressful conditions, which in turn triggers the activation of mTORC1 [2]. In addition, radiation-induced *DYRK3* expression promotes mitochondrial fission by mTORC1-dependent DRP1 activation, resulting in the promotion of brain tumors such as glioblastoma multiform [4]. However, DYRK3 might have opposing effects in mediating the balance between cell survival and death. For example, DYRK3 acts as a tumor suppressor in other cancers such as hepatocellular carcinoma [29]. In the present study we demonstrated that DYRK3 promoted cell viability in a condition of serum deprivation in HEK293 and H19-7 cells, which mainly occurred through SNAPIN phosphorylation and subsequent modulation of the SNAPIN-dynein and SNAPIN-synaptotagmin-1 interactions.

Depending on the cellular context, the exposure of cells to a serum-deficient environment stimulates multiple signaling pathways that affect cell death and survival, such as apoptosis, the UPS, and the autophagy lysosomal pathway (ALP). In addition, serum deprivation is widely used to synchronize cells at the G0/G1 phase by arresting the cell cycle [30]. Here, we found that serum deprivation caused a rapid decrease in DYRK3 expression in mammalian cells, reaching complete loss within 30 min. In our system, serum deprivation induced the rapid degradation of DYRK3 via ALP, but not via the UPS or caspase-dependent cell death pathway. This finding was further supported by an additional experiment to assess and compare the effect of serum derivation on DYRK3 degradation in *Atg5*-knockout MEFs. Among the ATG proteins, ATG5 is essential for autophagic vesicle formation, and is therefore one of the most commonly targeted genes in autophagy gene editing assays [31]. Therefore, ALP activity was downregulated or completely inhibited in *Atg5*-knockout cells. Applying this cell line to our experiment showed that DYRK3 was not completely degraded in *Atg5*-knockout MEFs, whereas it was completely degraded in control MEFs cultured in 5% FBS-containing medium.

In support of our results, several previous reports have shown ALP activation following serum deprivation. For example, serum deprivation-induced endosomal microautophagy plays a key role in regulating acute autophagic responses [32]. In addition, amino acid deprivation causes the endocytosis of specific membrane receptors, including p62 and NCOA4, and endosomal proteins, including NDP52 [33]. Interestingly, we found that serum deprivation-induced diminution of DYRK3 levels was not completely recovered in the presence of an autophagy inhibitor, indicating that additional mechanisms are at play in lowering DYRK3 levels under serum deprivation. This suggests that serum deprivation negatively affects the gene expression of *DYRK3*. Consistent with our data, the stability of various mRNA transcripts, including poly(A)-specific ribonuclease, in mammalian cell lines was previously reported to be reduced under serum deprivation conditions [34]. Taken together, our results highlight that among various toxic stimuli, serum deprivation specifically rapidly decreased DYRK3 levels via activation of the ALP pathway to promote DYRK3 degradation and the suppression of *Dyrk3* mRNA level.

In the current study SNAPIN was identified as a novel substrate of DYRK3 by yeast two-hybrid screening. SNAPIN is critical for neuron homeostasis through its two functional roles in the retrograde transfer of degradation targets, such as damaged mitochondria, autophagosomes, and mitophagosomes, and in the synaptic release of neurotransmitters [35, 36]. We demonstrated that the DYRK3-mediated phosphorylation of SNAPIN increased retrograde transport as well as neurotransmitter release. Comparing the biochemical and functional effects of wild-type SNAPIN and its phospho-mimetic mutant, endogenous SNAPIN was considered to be constitutively and sufficiently phosphorylated at Thr-14, maintaining the robust binding of SNAPIN to dynein and synaptotagmin-1, consequently achieving efficient axonal retrograde transport as well as synaptic neurotransmission. However, blockade of phosphorylation using SNAPIN-T14A mutant greatly suppressed these two regulatory roles of SNAPIN.

Axonal transport is the process by which neuronal cells transfer substrates between the cell body and the axon terminal [37] and operates in two directions: anterograde and retrograde transport. Two different motor proteins mediate this process: kinesin regulates anterograde transport, and dynein controls retrograde transport. In a previous report, SNAPIN was shown to be regulated by ALP degradation [38]. Here, we provide evidence that retrograde transport is modulated by SNAPIN phosphorylation and a subsequent increase in the interaction between SNAPIN and dynein. Defects in SNAPIN binding to dynein and the resultant impairment of axonal retrograde transport have been reported to contribute to the pathogenesis of AD. Since proper and efficient dynein-SNAPIN-mediated retrograde transport regulates the removal of mitophagosome from axon terminals, synaptic accumulation of toxic mitophagosomes is a feature of AD mice, which is attributed to the alteration in the binding affinity of SNAPIN and dynein, and consequently defective retrograde transport. For instance, when SNAPIN were overexpressed in the hippocampal neurons of the hAPP-transgenic AD mouse model, SNAPIN-mediated retrograde transport was found to contribute the removal of BACE1 from the axon terminal toward the cell body, thereby decreasing synaptic Aβ level [13]. Similarly, defects in synaptic mitochondria have been linked to the early pathophysiology of AD [20].

SNAPIN is phosphorylated by several kinases, including LRRK2, protein kinase A, casein kinase 1δ, and p38α-MAPK [10, 39–41]. More specifically, phosphorylation of SNAPIN at Thr-117 by LRRK2 reduces its binding to the SNARE complex, including SNAP25, resulting in a defect in neurotransmitter release [10]. Here, we demonstrated that DYRK3 also phosphorylates SNAPIN at Thr-14. Compared with the effect of LRRK2, we revealed that phosphorylation of SNAPIN by DYRK3 at Thr-14 did not affect the binding of SNAPIN to SNAP25 but enhanced its binding to synaptotagmin-1 and consequently promoted neurotransmitter release. We assumed that the phosphorylation at residue S50 by PKA increases the binding affinity of SNAPIN to SNAP25 [39], but that phosphorylation at T14 by DYRK3 may differentially affect the interaction with synaptotagmin-1 more readily than SNAP25. Furthermore, DYRK3 positively regulates retrograde transport and promotes cell viability, in contrast to the cytotoxic role of p38-MAPK. In addition, a mechanism which is SNAPIN-Dynein mediated retrograde transport is critical for quality control of intracellular components and neuronal homeostasis, and is thus essential for neuronal survival and development [42, 20]. Consequently, proper axonal retrograde transport through the DYRK3-SNAPIN-dynein interaction contributes to cell survival, which was significantly diminished under serum deprivation and thus greatly contributed to the toxicity of serum deprivation (Fig. 6 and Supplementary Fig. S4).

Collectively, these data suggest that DYRK3, as an upstream modulator of SNAPIN-dynein-mediated retrograde transport, might be a novel therapeutic target for neurotoxicity observed in AD pathology. Further studies are required to examine whether DYRK3-mediated phosphorylation of SNAPIN could reduce the synaptic production of Aβ, and attenuate the synapse loss and/or cognitive deficits seen in patients with AD.

## Supplementary information

Supplementary methods

Supplementary data

Original Data File

## Data Availability

The data used and analyzed during the current study are available from the corresponding author on reasonable requests.
