# Supplementary material for: DYRK3 phosphorylates SNAPIN to regulate axonal retrograde transport and neurotransmitter release
Source: Cell Death Discov. 2022 Dec 30;8:503. doi: 10.1038/s41420-022-01290-0 (PMC9803678; doi:10.1038/s41420-022-01290-0)
Supplement: Supplementary file 1 — Supplementary methods [file 41420_2022_1290_MOESM1_ESM.docx]

**Supplementary Materials and Methods**

*Materials* **-** Dulbecco's modified Eagle medium (DMEM), fetal bovine serum (FBS), Alexa Fluor 488-conjugated mouse IgG (A-11029), Alexa Fluor 594-conjugated rabbit IgG (A-11012), ProLong Gold Antifade Mountant with 4′,6-diamidino-2-phenylindole (DAPI) (P-36931), and anti-V5 (R960-25) antibodies were purchased from Invitrogen (Carlsbad, CA, USA). Protein A-Sepharose beads and Ni-NTA agarose beads were purchased from GE Healthcare Life Sciences (Piscataway, NJ, USA). Enhanced chemiluminescence (ECL) reagents were purchased from AbClon (Seoul, Korea). Mouse monoclonal anti-Flag(F3165-1MG) antibody and polyethylenimine were purchased from Sigma-Aldrich (St. Louis, MO, USA). Horseradish peroxidase-conjugated anti-rabbit and anti-mouse secondary antibodies were purchased from EMD Millipore (Billerica, MA, USA). Rabbit anti-pThr (ab9337) and polyclonal anti-V5 (ab9116) antibodies were purchased from Abcam (Cambridge, UK). Mouse monoclonal anti-DYRK3 (sc-390532), rabbit polyclonal anti-Flag (F7425-2MG), anti-actin, anti-Hsp90 (sc-13119), mouse anti-GFP (sc-9996), and anti-tubulin (sc-8017) antibodies were purchased from Santa Cruz Biotechnology (Santa Cruz, CA, USA). Polyclonal anti-HA (PAB0861) and anti-Myc (PAB10345) antibodies were obtained from Abnova (Taipei, Taiwan). Mouse monoclonal anti-HA (ab9337) and rabbit anti-LC3 (#2775) antibodies were purchased from Covance (Princeton, NJ, USA) and Cell Signaling (Danvers, MA, USA), respectively. Rabbit polyclonal anti-SNAPIN (10055-1-AP) antibodies were purchased from Proteintech Group (Rosemont, IL, USA).

*DNA constructs* **-** Mammalian constructs encoding N-terminally Flag-tagged human wild-type DYRK3 (pRK5-Flag-DYRK3-WT) and its kinase-inactive mutant with the K238M substitution (pRK5-Flag-DYRK3-KM) were prepared using the QuikChange XL site-directed mutagenesis kit (Agilent Technologies, Santa Clara, CA, USA). Plasmids encoding either Myc- (pcDNA3.1-Myc-SNAPIN) or His-tagged wild-type SNAPIN (pET29b-His-SNAPIN) and HA-tagged SNAP25 (pcDNA3.1-HA-SNAP25) were kindly provided by W. Seol (Wonkwang University School of Medicine, Gunpo-si, Kyeonggi-do, Korea). Plasmid encoding GFP-tagged wild-type dynein (pEFGP-Dynein-IC2-FL) was kindly provided by M.J. Lee (Seoul National University, Seoul, Korea). Plasmids encoding HA-tagged wild-type dynein A (dynein cytoplasmic 1 intermediate chain 1, pSG5-HA-Dynein A) and wild-type dynein B (dynein cytoplasmic 1 intermediate chain 2, pSG5-HA-Dynein B) were kindly provided by K. Ahn (Seoul National University, Seoul, Korea). The mammalian construct encoding EGFP-tagged human SNAPIN (pEGFP-SNAPIN) was purchased from ADDGENE. To generate SNAPIN constructs encoding a variety of point-mutants with substitution of single or double amino acids, such as S6/S10A, T14/T20A, S42/S50A, T63A, S112/T117A, S126/S133A, T14A, or T14E, site-directed mutagenesis reactions were performed using the QuikChange XL site-directed mutagenesis kit. All cDNA constructs were confirmed using DNA sequencing (BIONICS, Seoul, Korea).

*Yeast two-hybrid assay* **-** For yeast two-hybrid assays, we utilized the Matchmaker Gold System (Clontech, Mountain View, CA, USA) and the Mate & Plate library of the human brain (Clontech) which was already transformed into yeast strain Y2H Gold. The bait plasmid encoding DYRK3 was generated into pGBKT7 by polymerase chain reaction (PCR) amplification using the forward primer 5′-CCGAATTCATGGGAGGCACAGCTC-3′ and the reverse primer 5′-CTGCTGCAGCTAGCTAATCAGTTTTGGCAAT-3’. The expression of the bait vector, cell lysis, and immunoblot analysis were performed according to the manufacturer's instructions. The prey vectors from the library and bait plasmid were co-transformed into a yeast strain Y2HGold having a *MATa* GAL4 reporter for screening, and positive clones were selected by growth on SD/-Leu/-Trp media. Veriﬁed clones were then selected on SD/-Ade/-His/-Leu/-Trp media, stained with X-α-Gal to determine β-galactosidase activity, and verified by PCR amplification. Finally, the candidate protein(s) in the prey vectors that passed through multiple selection processes was identified by DNA sequencing using the T7 sequencing primer.

*Purification of bacterial recombinant SNAPIN and synpatoatgmin-1 protein* ***-*** *Escherichia coli* BL21(DE3)-Codon Plus competent cells (Invitrogen) were transformed with the bacterial plasmid encoding either His-tagged SNAPIN (pET28b-His-SNAPIN) or synaptotagmin-1 (pET28b-His-SNAPIN) for 24 h and incubated with 0.5 or 1 mM isopropyl-β-D-1-thiogalactopyranoside for an additional 2 or 4 h at 37^o^C, respectively. Cells were resuspended in lysis buffer (50 mM Tris-HCl, pH 7.5, 1% NP40, 1% Triton-X100, 10% glycerol, 150 mM NaCl, and 20 mM imidazole) and incubated for 20 min at 4^o^C. Cell lysates were then incubated for 3 h with Ni-NTA resin and washed three times with the same buffer containing 100 mM imidazole. The resin-bound proteins were eluted with elution buffer containing 500 mM imidazole, followed by sodium dodecyl sulfate-polyacrylamide gel electrophoresis (SDS-PAGE). Bacterial recombinant DYRK3 protein was kindly provided by H.S. Cho (Yonsei University, Seoul, Korea).

*Co-immunoprecipitation and western blot analysis* **-** Cell lysates were prepared by rinsing cells with ice-cold phosphate-buffered saline (PBS) and lysed with 1% Nonidet P-40 lysis buffer (50 mM Tris, pH 7.5, 150 mM NaCl, 1% Nonidet P-40, 10% glycerol, 0.2 mM phenylmethylsulfonyl fluoride, 1 mM Na_3_VO_4_, 10 mM NaF, and 1x protease inhibitor cocktail [including 1 μg/ml aprotinin, 1 μg/ml leupeptin, and 1 μg/ml pepstatin]). The cells were then scraped, and supernatants were collected after centrifugation at 15,700 x *g* for 15 min at 4°C. For immunoprecipitation, 1 μg of the appropriate antibody was incubated with 1,000 μg of the cell lysate overnight at 4°C with gentle rotation. The mixture was then incubated with 30 μl of a 1:1 mixture of the protein A-Sepharose bead suspension for 2 h at 4°C. The beads were pelleted by centrifugation at 9,300 x *g* for 1 min and washed three times with 1% Nonidet P-40 lysis buffer. The immunocomplexes were boiled in SDS-PAGE sample buffer, separated by SDS-PAGE, and transferred to nitrocellulose membranes (Millipore, Burlington, MA, USA). The membranes were blocked for 1 h at room temperature using Tris-buffered saline with Tween^®^ (TBST; 25 mM Tris, pH 7.5, 150 mM NaCl, and 0.1% Tween^®^20) containing 5% nonfat dry milk and then incubated overnight at 4°C in TBST containing the appropriate antibody. The membranes were washed three times in TBST and incubated with the secondary antibody (horseradish peroxidase-conjugated IGG antibody) for 2 h. The blots were washed three times with TBST for 10 min, and the bands were visualized using ECL reagents (Abclon, Seoul, Korea) following the manufacturer’s instructions.

*Preparation of cytosolic and nuclear fractions* **-** Cells were scraped in ice-cold PBS and lysed with hypotonic buffer (10 mM HEPES, pH 7.9, 1.5 mM MgCl_2_, 10 mM KCl, 0.5 mM dithiothreitol [DTT], and protease inhibitor cocktail). The cells were incubated for 10 min on ice and then added with 1% NP-40 by vortex for 5 sec. The cell lysates were centrifuged at 13,000 x g for 5 min at 4°C. The supernatants were collected as the cytosolic fraction. The nuclear pellet was washed with hypotonic buffer and resuspended in hypertonic buffer (27 mM HEPES, pH 7.9, 2 mM MgCl_2_, 560 mM NaCl, 270 mM EDTA, 33% glycerol, 0.5mM DTT, and protease inhibitor cocktail). The pellets were lysed using 1% NP-40 and incubated for 20 min on ice, followed by centrifugation at 13,000 × g for 20 min at 4 °C. The supernatants were collected as the nuclear fraction.

*RNA extraction and reverse transcription real-time PCR analysis* **-** After cells were incubated with DMEM for 30 or 60 min at 37°C, total RNAs were extracted with Trizol reagent, followed by cDNA synthesis using the Primescript RT master Mix kit (TAKARA). The cDNA was incubated with SYBR Green Real-time PCR master mix (Toyobo Co. Ltd., Osaka, Japan) containing 10 pg/ml of the forward and reverse primers, and amplified using a Light Cycler PCR system (Roche Applied Sciences, Indianapolis, IN, USA). The primer sequences of *DYRK3* used were 5′-CCCTCTGCCCGCTTGAC-3′ (forward) and 5′- CCCGTTTCCCTGACACCTT-3′ (reverse) to generate the PCR products. *GAPDH* was used as a control, using the forward primer, 5′-CCCGTTTCCCTGACACCTT-3′ and the reverse primer, 5′- CATACCAGGAAATGAGCTTG-3′ to generate PCR products (1).

*In vitro kinase assay* **-** After HEK293 cells were transfected for 24 h with plasmids encoding Myc-tagged SNAPIN-WT or one of its point-mutants, the cells were lysed in 1% NP-40 lysis buffer. Cell lysates were then immunoprecipitated overnight at 4°C with anti-Myc antibodies, and anti-Myc immunocomplexes were used as the source of SNAPIN in the assay. In the same way, anti-immunocomplexes of SNAP25 and dynein were produced from the mammalian constructs encoding SNAP25 and dynein with different N-terminal tags produced, such as HA-SNAP25 and GFP-Dynein, respectively, which were utilized as another substrate source. For the kinase assay, the immunocomplexes obtained from Myc-tagged SNAPIN, HA-tagged SNAP25, or GFP-Dynein were mixed with 1 μg of bacterial recombinant DYRK3 protein and 1x reaction buffer containing 0.2 mM Na_3_VO_4_ and 10 μM ATP. The *in vitro* kinase reaction was initiated by the addition of 10 μCi [γ-^32^P]ATP, allowed to proceed for 30 min at 30°C, and terminated by the addition of SDS-PAGE sample buffer. The samples were resolved by SDS-PAGE and the incorporated [γ-^32^P]ATP radioisotope was detected by autoradiography.

*Analysis of serum deprivation-induced cell death in H19-7 and HEK293 cells* **-** Cellular cytotoxicity was measured using the LDH Cytotoxicity Detection Kit (Takara Korea, Seoul, Korea), according to the manufacturer’s protocol. To assess the serum deprivation-induced cytotoxicity in H19-7 and HEK293 cells, DMEM was added to the cells before reaching 70-90% confluence, and the cells were cultured at 37°C for 24 h. The supernatant (100 µl) was then transferred to a new 96-well plate, and 100 µl of the lactate dehydrogenase (LDH) substrate mix was added. After 20-min incubation at room temperature, the absorbance of the sample was measured at 490-600 nm.

*Confocal microscopic analysis* **-** HEK293 cells were seeded onto poly-L-lysine-coated coverslips in 6-well plates to approximately 50-70% confluence at 37°C. After DNA transfection for 24 h, the cells were washed twice with PBS (pH 7.4), fixed with 7.3% formaldehyde for 30 min, permeabilized with 0.2% Triton X-100 for 30 min, and blocked with 1% bovine serum albumin for 30 min at room temperature. The cells were then stained with anti-Flag or anti-Myc antibodies, and fluorescein isothiocyanate-conjugated secondary antibodies were used to detect the primary antibodies. The samples were counterstained with DAPI, mounted, and analyzed using an LSM 880 confocal microscopy (Carl Zeiss, Oberkochen, Germany). The data were processed by Zeiss LSM Image Browser (Carl Zeiss).

*Rat hippocampal neuron culture and DNA transfection* **-** Rat hippocampal primary neurons were derived from embryonic day 18 Sprague Dawley fetal rats. The hippocampi were dissociated with papain and triturated with a polished half-bore Pasteur pipette. Dissociated neurons (2.5 x 10^5^) in minimum Eagle’s medium (MEM; Invitrogen) supplemented with 0.6 % glucose, 1 mM pyruvate, 2 mM L-glutamate, 10 % FBS (Hyclone) were plated on poly-D-lysine-coated 18 mm glass coverslips in a 60 mm petri dish. Three hours after plating, the medium was replaced with neurobasal media (Invitrogen) supplemented 2 % B-27 and 0.5 mM L-glutamate. Cultured neurons were transfected using the calcium-phosphate method. Six micrograms of cDNA with small interfering RNA (siRNA) and 9.3 μl of 2 M CaCl_2_ were mixed in distilled water to a total volume of 75 μl, and the same volume of 2x borate buffered saline was added. The cell culture medium was replaced by transfection medium (MEM, 1 mM pyruvate, 0.6 % glucose, 10 mM glutamine, and 10 mM HEPES, pH 7.7). Subsequently, the cDNA mixture was added and the neurons were incubated in a 37℃ CO_2_ incubator for 60 min. The transfection medium was replaced by washing medium (MEM, 1 mM pyruvate, 0.6 % glucose, 10 mM glutamine, and 10 mM HEPES, pH 7.4), and the neurons were incubated in 37℃ CO_2_ incubator for 30 min. The washing medium was then replaced with the original culture medium.

*Assay of synaptic vesicle exocytosis and endocytosis* **-** Transfected neurons on coverslips were mounted in a perfusion/stimulation chamber equipped with platinum-iridium field stimulus electrodes (Chamlide, LCI) on the stage of an Olympus IX-71 inverted microscope with 40x 1.0 N.A. oil lens. Coverslips were continuously perfused at 35^o^C with Tyrode’s solution supplemented 10 μM 6-cyano-7-nitroquinoxaline-2,3-dione (CNQX) and 50 μM DL-2-amino-5-phosphonovaleric acid (AP-V) to prevent recurrent neuronal excitation during stimulation. Time-lapse images were acquired every 5 sec for 5 min using an Andor Zyla-5.5-CL3 sCMOS camera (Andor Technologies) driven by MetaMorph imaging software (Universal Imaging Corporation). From the fifth frame, the neurons were stimulated (1 msec, 20-50 V, bipolar, total 900 action potentials [APs], 20 Hz) using an A310 Accupulser current stimulator (World Precision Instrument). Quantitative measurements of the fluorescent intensity at individual boutons were obtained by averaging the selected area of pixel intensities by using Fiji/ImageJ software. Individual regions were selected manually, circular regions of interest were drawn around the synaptic boutons, and the average intensities were calculated. The fluorescence intensity was normalized from 0 to 1, which corresponds to the fluorescence values averaged over all pixels within the region of interest. Single-exponential fitting was performed using Origin 9 (OriginLab) to obtain the decay time constant (tau).

**References**

1. Kim K, Lee S, Kang H, Shin E, Kim HY, Youn H, Youn B. Dual Specificity Kinase DYRK3 Promotes Aggressiveness of Glioblastoma by Altering Mitochondrial Morphology and Function. *Int J Mol Sci* **22**, 2982 (2021)
