# Supplementary material for: DYRK3 phosphorylates SNAPIN to regulate axonal retrograde transport and neurotransmitter release
Source: Cell Death Discov. 2022 Dec 30;8:503. doi: 10.1038/s41420-022-01290-0 (PMC9803678; doi:10.1038/s41420-022-01290-0)
Supplement: Supplementary file 2 — Supplementary data [file 41420_2022_1290_MOESM2_ESM.pdf]

Supporting information

DYRK3 phosphorylates SNAPIN to regulate axonal retrograde transport and neurotransmitter release

Ye Hyung Lee, Bo Kyoung Suh, Unghwi Lee, Seung Hyun Ryu, Sung Ryong Shin, Sunghoe Chang, Sang Ki Park, and Kwang Chul Chung

Contents

1. **Supplementary Figure S1.** Serum deprivation causes the rapid reduction of intracellular DYRK3 levels.
2. **Supplementary Figure S2.** Serum deprivation triggers the degradation of DYRK3 protein via autophagy-lysosome pathway as well as the decrease of Dyrk3 mRNA level.
3. **Supplementary Figure S3.** The phosphorylation of SNAPIN by DYRK3 enhances its interaction with synaptotagmin-1, but not with SNAP25, in the SNARE complex.
4. **Supplementary Figure S4.** Working model of DYRK3-SNAPIN-mediated regulation of retrograde axonal transport and synaptic neurotransmitter release in the resting state and their effects on cell death under serum deprivation.

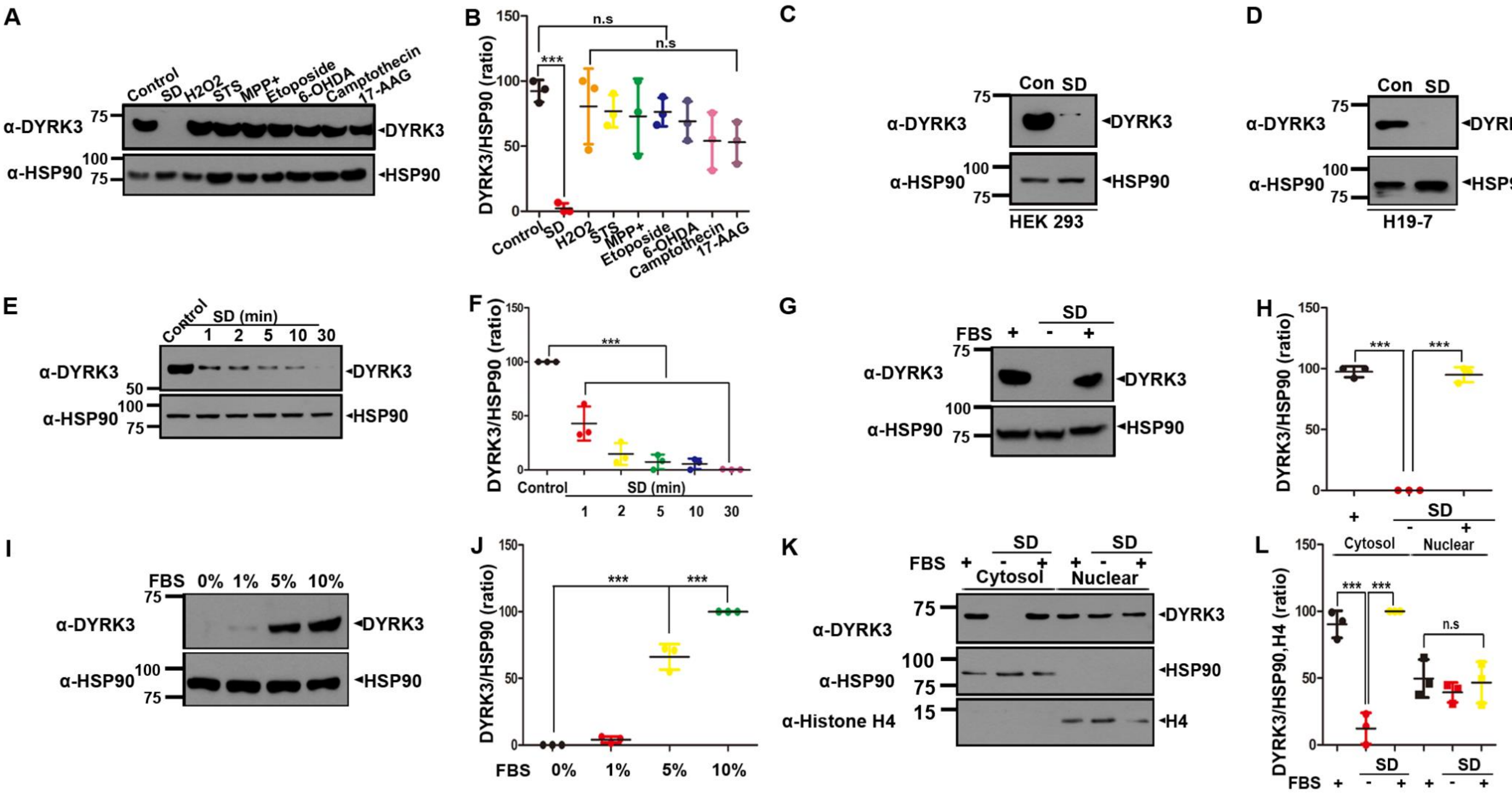
A
Control
SD
H2O2
STS
MPP+
Etoposide
6-OHDA
Camptothecin
17-AAG
α-DYRK3
α-HSP90
DYRK3
HSP90
B
DYRK3/HSP90 (ratio)
n.s
***
C
Con SD
HEK 293
D
H19-7
E
SD (min)
1 2 5 10 30
F
G
FBS
H
I
FBS 0% 1% 5% 10%
J
K
Cytosol
Nuclear
α-Histone H4
H4
L
DYRK3/HSP90,H4 (ratio)

Supplementary Figure S1. Serum deprivation causes the rapid reduction of intracellular DYRK3 levels. (A) HEK293 cells were left untreated (Control) or treated for 6 h with DMEM (SD, serum deprivation), H2O2 (200 μM), staurosporine (STS, 1 μM), MPP+ (500 μM), etoposide (100 μM), 6-OHDA (200 μM), camptothecin (1 μM), or 17-AAG (1 μM), as indicated. Cell lysates were immunoblotted with anti-DYRK3 antibodies; Hsp90 served as a loading control. **(B)** Quantitation of expression in the blots shown in (A). All data represent the mean ± standard deviation of three independent experiments (***p < 0.001; n.s., not significant). **(C, D)** After HEK293 (C) or H19-7 cells (D) were left untreated (Control) of treated for 6 h with DMEM (SD), cell lysates were immunoblotted with anti-DYRK3 antibodies. **(E)** After HEK293 cells were incubated with DMEM for the indicated times, cell lysates were immunoblotted with anti-DYRK3 antibody. **(F)** Quantification of expression levels from the blots in (E). All data represent the mean ± standard deviation of three independent experiments (***p < 0.001). **(G)** HEK293 cells were left untreated or treated for 6 h with DMEM and then the medium was replaced with 10% FBS-containing DMEM for further culture of 2 h. Cell lysates were immunoblotted with anti-DYRK3 antibodies. **(H)** Quantification of expression levels from the blots in (G). All data represent the mean ± standard deviation of three independent experiments (***, p < 0.001). **(I)** Where indicated, HEK293 cells were left treated for 6 h with 0, 1, 5, or 10% FBS-containing DMEM. Cell lysates were immunoblotted with anti-DYRK3 antibodies. **(J)** Quantification of the expression levels from the blots in (I). All data represent the mean ± standard deviation of three independent experiments (***p < 0.001). **(K)** HEK293 cells were left untreated or treated for 6 h with DMEM and then the medium was replaced with 10% FBS-containing DMEM for further culture of 2 h. Cell lysates were fractionated into cytosolic and nuclear fractions. Each fraction was immunoprecipitated with anti-DYRK3 antibody, followed by immunoblotting with the indicated antibodies. Histone H4 and Hsp90 served as the markers for the nuclear and cytosolic fraction, respectively. **(L)** Quantification of expression levels in the blots of (K). All data represent the mean ± standard deviation of three independent experiments (***p < 0.001).

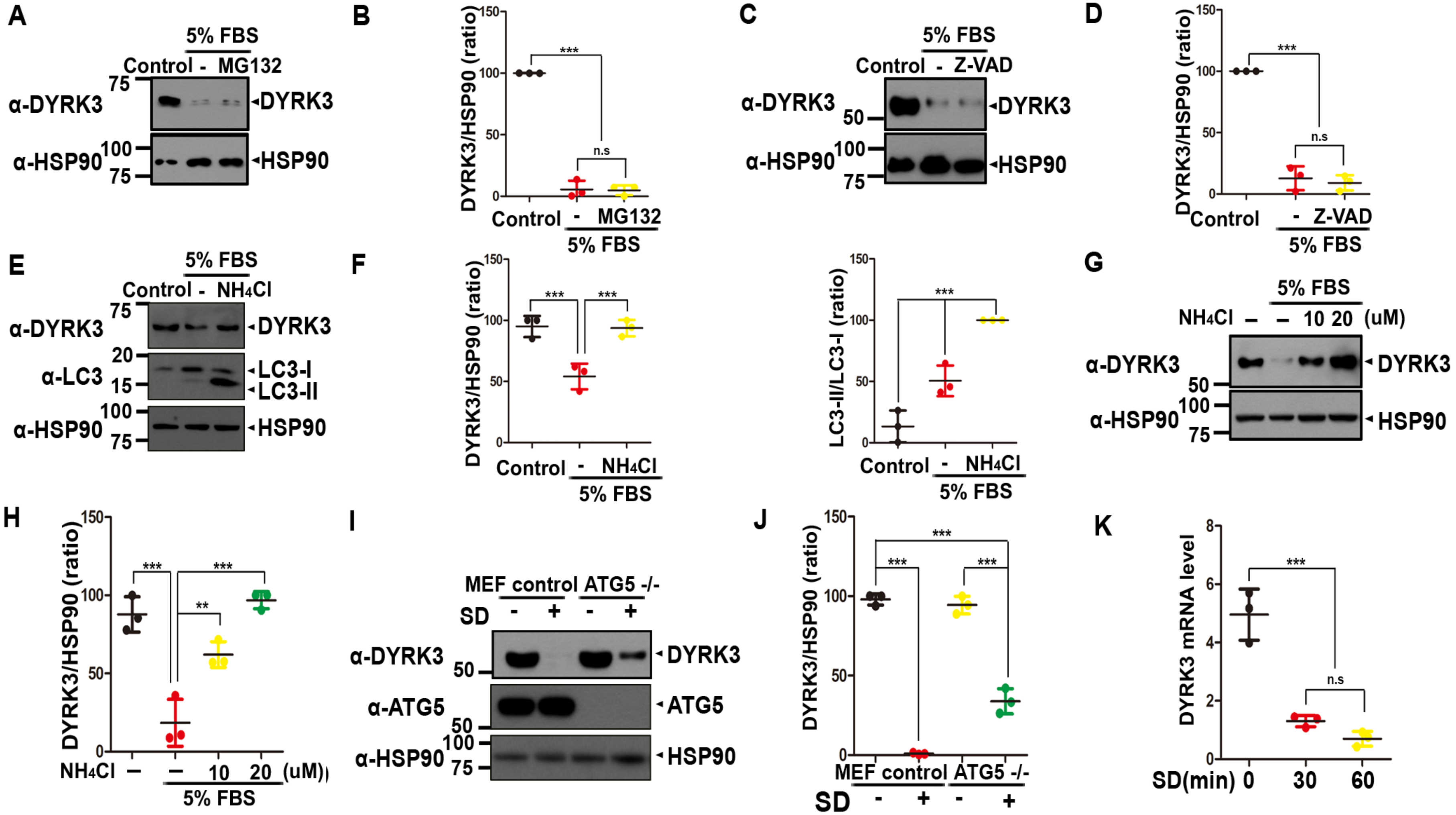
A
5% FBS
Control - MG132
α-DYRK3
DYRK3
α-HSP90
HSP90
B
DYRK3/HSP90 (ratio)
Control - MG132
5% FBS
C
5% FBS
Control - Z-VAD
α-DYRK3
DYRK3
α-HSP90
HSP90
D
DYRK3/HSP90 (ratio)
Control - Z-VAD
5% FBS
E
5% FBS
Control - NH4Cl
α-DYRK3
DYRK3
α-LC3
LC3-I
LC3-II
α-HSP90
HSP90
F
DYRK3/HSP90 (ratio)
Control - NH4Cl
5% FBS
LC3-II/LC3-I (ratio)
Control - NH4Cl
5% FBS
G
5% FBS
NH4Cl – – 10 20 (uM)
α-DYRK3
DYRK3
α-HSP90
HSP90
H
DYRK3/HSP90 (ratio)
NH4Cl – – 10 20 (uM)
5% FBS
I
MEF control ATG5 -/-
SD - + - +
α-DYRK3
DYRK3
α-ATG5
ATG5
α-HSP90
HSP90
J
DYRK3/HSP90 (ratio)
MEF control ATG5 -/-
SD - + - +
K
DYRK3 mRNA level
SD(min) 0 30 60

Supplementary Figure S2. Serum deprivation triggers the degradation of DYRK3 protein via autophagy-lysosome pathway as well as the decrease of Dyrk3 mRNA level. (A, C, E) Where indicated, HEK293 cells were left untreated (Control) or treated for 2 h with DMEM containing 5% FBS and treated for an additional 6 h with vehicle (-), 10 μM MG132 (A), 10 μM Z-VAD (C), or 10 μM NH_4Cl. Cell lysates were immunoblotted with anti-DYRK3 (A, C, E) or anti-LC3 (E) antibody. Hsp90 served as a loading control. **(B, D, F)** Quantification of expression levels in the blots in (A, C, E). Data represent the mean ± standard deviation of three independent experiments (***$p < 0.001$; n.s., not significant). **(G)** Where specified, HEK293 cells were left untreated (Control) or treated for 2 h with DMEM containing 5% FBS and treated for an additional 6 h with vehicle (−) or NH_4Cl (10 or 20 μM). Cell lysates were immunoblotted with anti-DYRK3 antibodies. **(H)** Quantification of the expression levels in the blots of (G). All data represent the mean ± standard deviation of three independent experiments (**$p < 0.01$, ***$p < 0.001$). **(I)** Control MEF and *Atg5*-knock out MEFs were left untreated (-) or treated for 6 h with DMEM containing 5%. Cell lysates were immunoblotted with anti-DYRK3 antibody. **(J)** Quantification of expression levels in the blots of (I). All data represent the mean ± standard deviation of three independent experiments (***$p < 0.001$). **(K)** Where indicated, HEK293 cells were treated for the indicated times with DMEM. Total RNA was extracted from with Trizol reagent and reverse transcribed. Levels of *Dyrk3* mRNA were measured using real-time PCR. All data represent the mean ± standard deviation of three independent experiments (***$p < 0.001$; n.s., not significant).

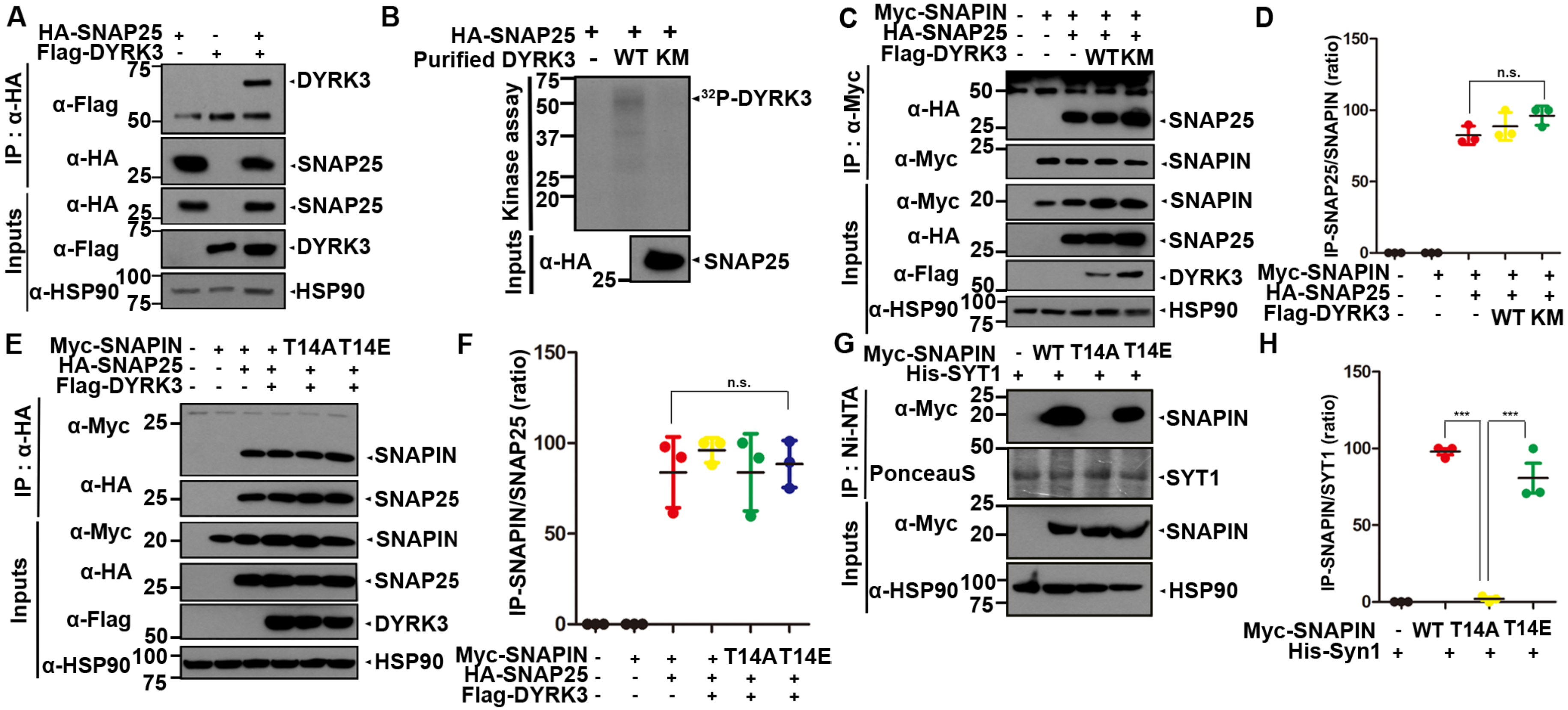

A
HA-SNAP25
Flag-DYRK3
IP : α-HA
Inputs
α-Flag
α-HA
α-HSP90
DYRK3
SNAP25
HSP90
B
HA-SNAP25
Purified DYRK3
WT KM
Kinase assay
32P-DYRK3
SNAP25
C
Myc-SNAPIN
IP : α-Myc
α-Myc
SNAPIN
D
IP-SNAP25/SNAPIN (ratio)
n.s.
E
T14A T14E
F
IP-SNAPIN/SNAP25 (ratio)
G
His-SYT1
IP : Ni-NTA
PonceauS
SYT1
H
IP-SNAPIN/SYT1 (ratio)
***
His-Syn1

Supplementary Figure S3. The phosphorylation of SNAPIN by DYRK3 enhances its interaction with synaptotagmin-1, but not with SNAP25, in the SNARE complex. (A) After HEK293 cells were transfected for 24 h with a plasmid encoding HA-SNAP25 or Flag-DYRK3 alone or in combination, cell lysates were immunoprecipitated with anti-HA antibody, followed by immunoblotting with the indicated antibody. **(B)** After HEK293 cells were transfected for 24 h with a plasmid encoding HA-SNAP25, cell lysates (~ 1,000 μg of protein) were immunoprecipitated with anti-HA antibody. For the in vitro kinase assay, the anti-HA immunocomplex as a substrate was mixed with bacterially expressed DYRK3-WT or DYRK3-KM, incubated for 30 min at 30oC with kinase buffer and [γ-32P]ATP, resolved by SDS-PAGE, and analyzed by autoradiography. Proper expression of transiently expressed SNAP25 in cell extracts was verified by western blotting with anti-HA antibody (Input). **(C)** HEK293 cells were transfected for 24 h with a plasmid encoding Myc-SNAPIN, HA-SNAP25, Flag-DYRK3-WT, or Flag- DYRK3-KM alone or in combination. Cell lysates were immunoprecipitated with anti-Myc antibody, followed by immunoblotting with the indicated antibody. **(D)** Quantification of the blots in (C). All data represent the mean ± standard deviation of three independent experiments (n.s., not significant). **(E)** HEK293 cells were transfected for 24 h with a plasmid encoding Myc-SNAPIN, Myc-SNAPIN-T14A, Myc-SNAPIN-T14E, HA-SNAP25, or Flag-DYRK3-WT alone or in combination. Cell lysates were immunoprecipitated with anti-HA antibody, followed by immunoblotting with the indicated antibody. **(F)** Quantification of the blots in (E). All data represent the mean ± standard deviation of three independent experiments (n.s., not significant). **(G)** After HEK293 cells were transfected for 24 h with a plasmid encoding Myc-SNAPIN, Myc-SNAPIN-T14A, or Myc-SNAPIN-T14E, cell lysates were immunoprecipitated with anti-Myc antibody. For the pull-down assay, bacterially expressed His-tagged synaptotagmin-1 (SYT1) bound to Ni-NTA Sepharose beads were incubated with anti-Myc immunoprecipitates overnight. Cell lysates were immunoblotted with anti-Myc antibody. The blots of SYT1 were subjected to Ponceau S staining. **(H)** Quantification of the blots in (G). All data represent the mean ± standard deviation of three independent experiments (***p < 0.001).

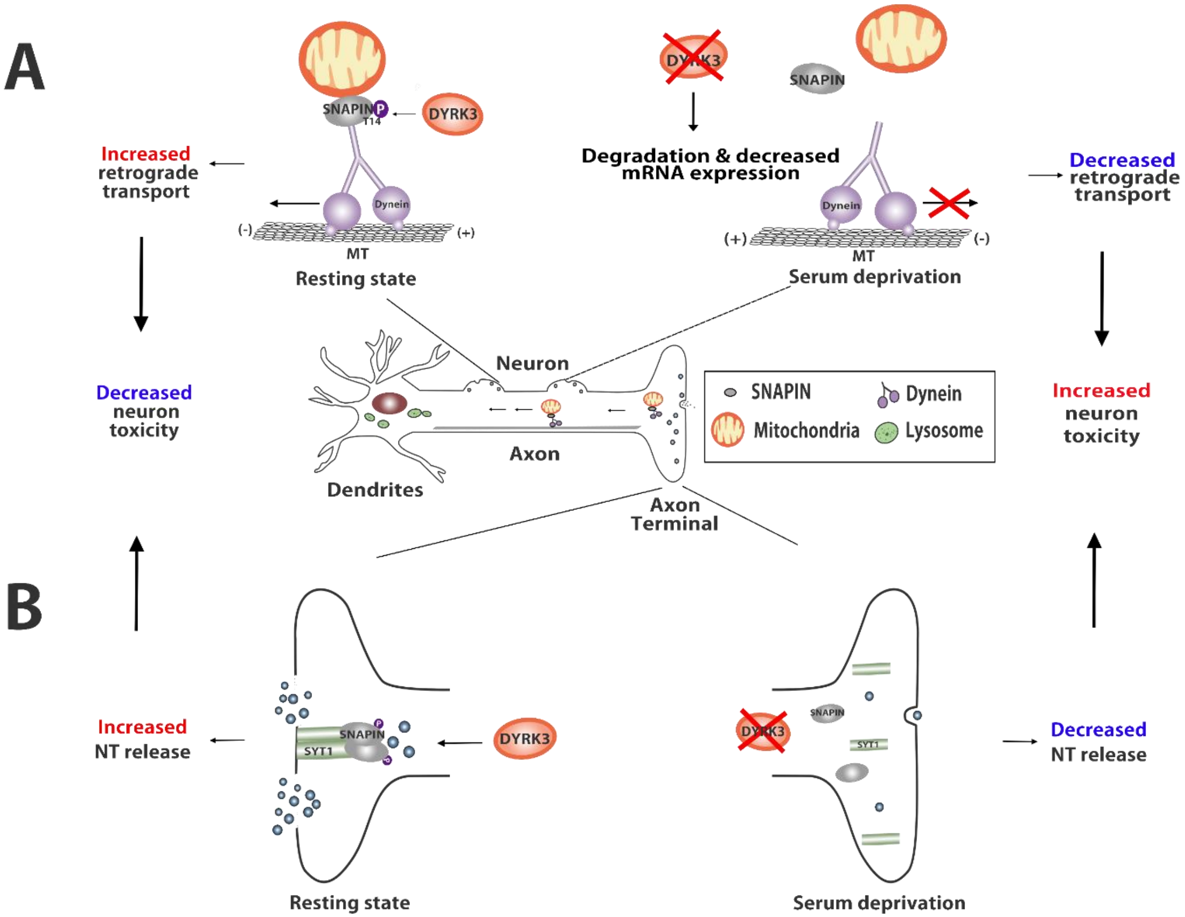

Supplementary Figure S4. Working model of DYRK3-SNAPIN-mediated regulation of retrograde axonal transport and synaptic neurotransmitter release in the resting state and their effects on cell death under serum deprivation. (A) In the normal and resting state, DYRK3 phosphorylates SNAPIN at threonine 14 in mammalian neuronal cells, which then increases the binding of SNAPIN to dynein and to synaptotagmin-1 (SYT1), and subsequently promotes retrograde axonal transportation and neurotransmitter release, respectively. These actions consequently contribute to the cytoprotective activity of DYRK3 in neurons. **(B)** Under serum deprivation, the intracellular DYRK3 level is rapidly reduced via autophagy protein degradation of DYRK3 and suppression of Dyrk3 mRNA transcription, leading to the blockade of the DYRK3-mediated phosphorylation of SNAPIN. This event then causes the separation of SNAPIN from dynein as well as from SYT1 composed of SNARE complex, and thus impaired SNAPIN-dynein-mediated retrograde transport and SNAPIN-SYT1-mediated neurotransmitter release, consequently increasing toxicity in neuronal cells.
