# Supplementary material for: DYRK3 phosphorylates SNAPIN to regulate axonal retrograde transport and neurotransmitter release
Source: Cell Death Discov. 2022 Dec 30;8:503. doi: 10.1038/s41420-022-01290-0 (PMC9803678; doi:10.1038/s41420-022-01290-0)

Original western blots

Figure 1.

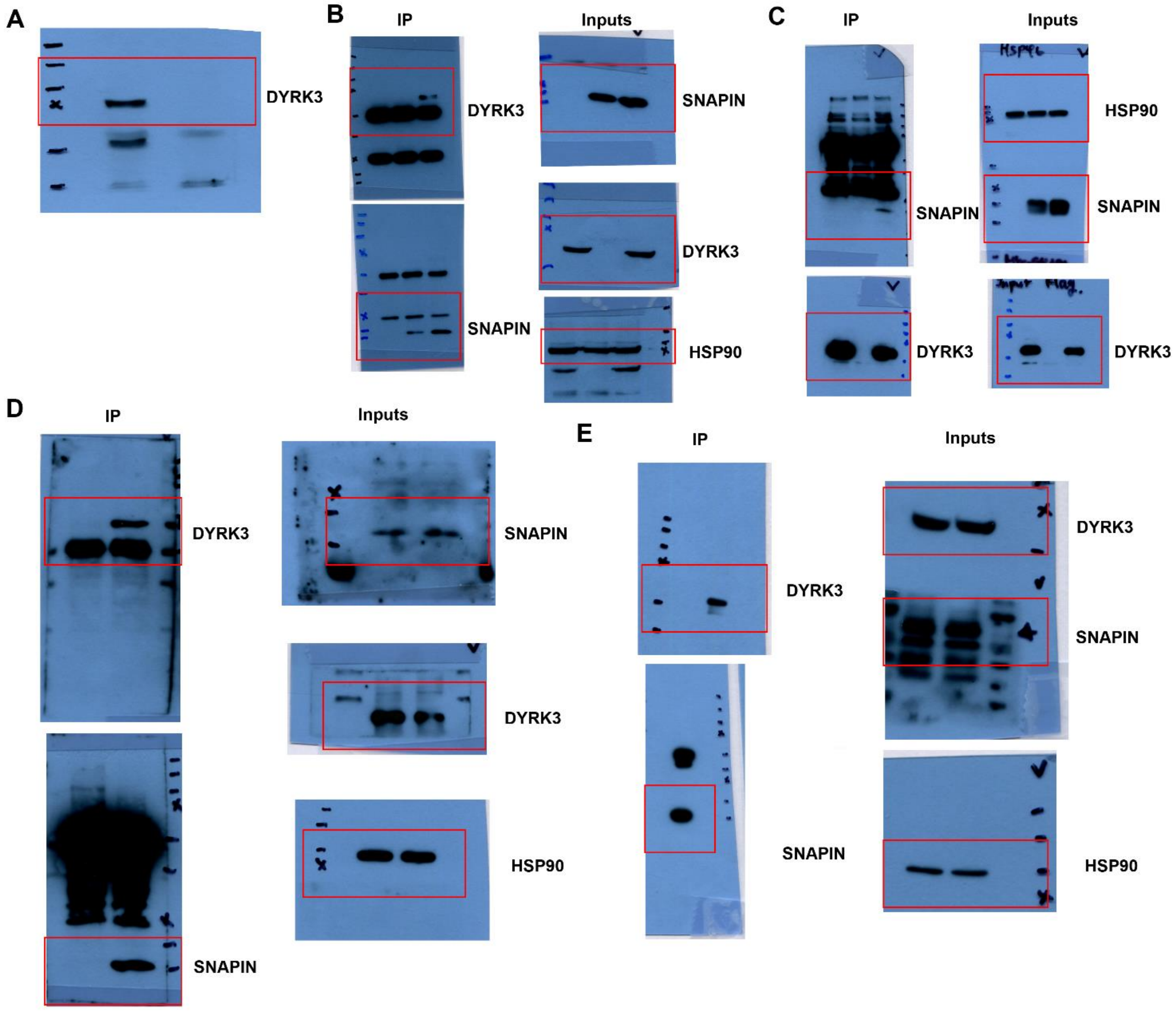

Figure 2.

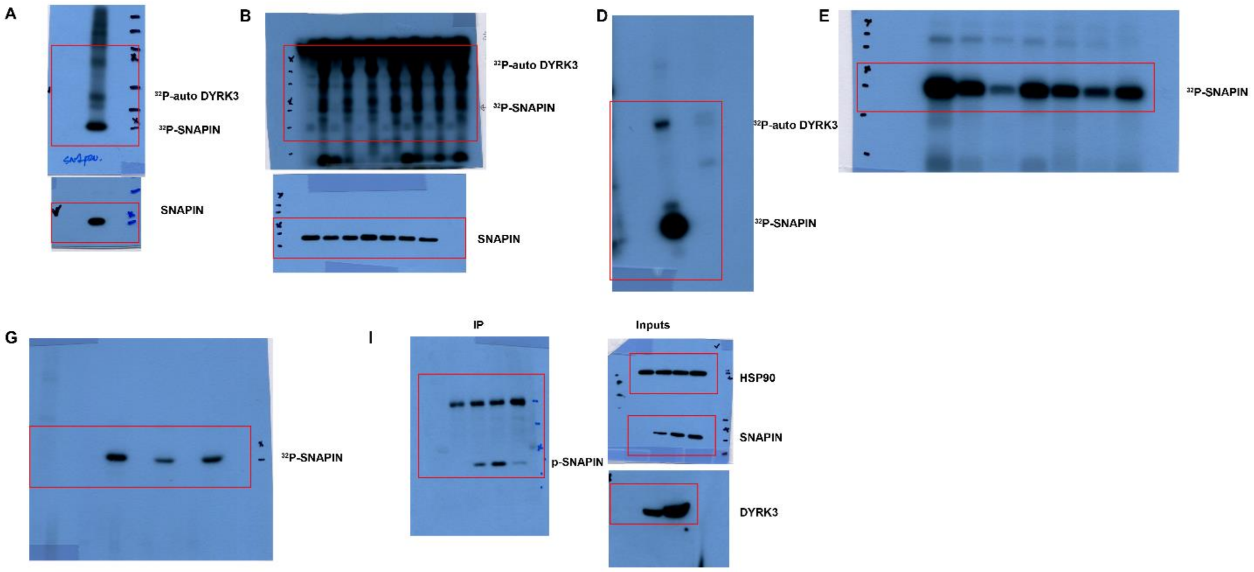

Figure 3.

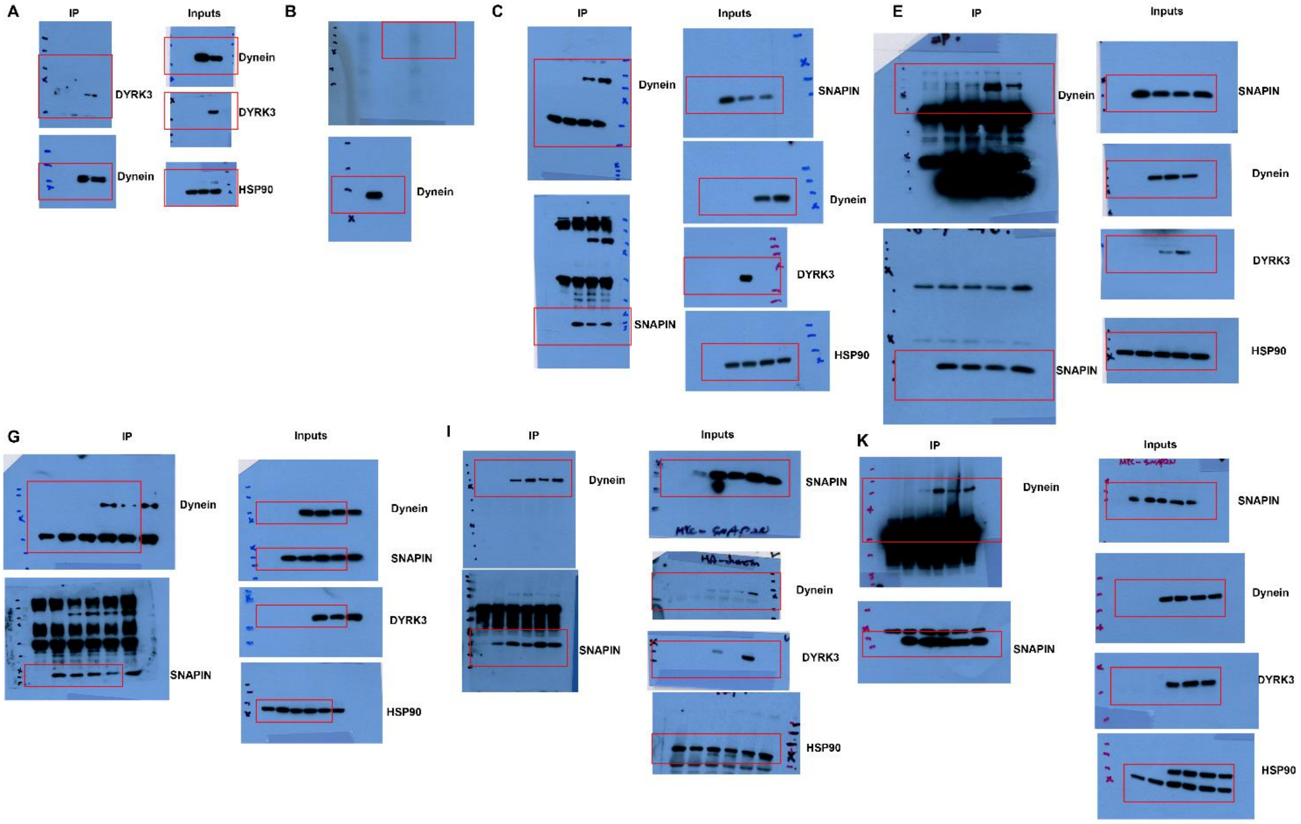

Figure 6.

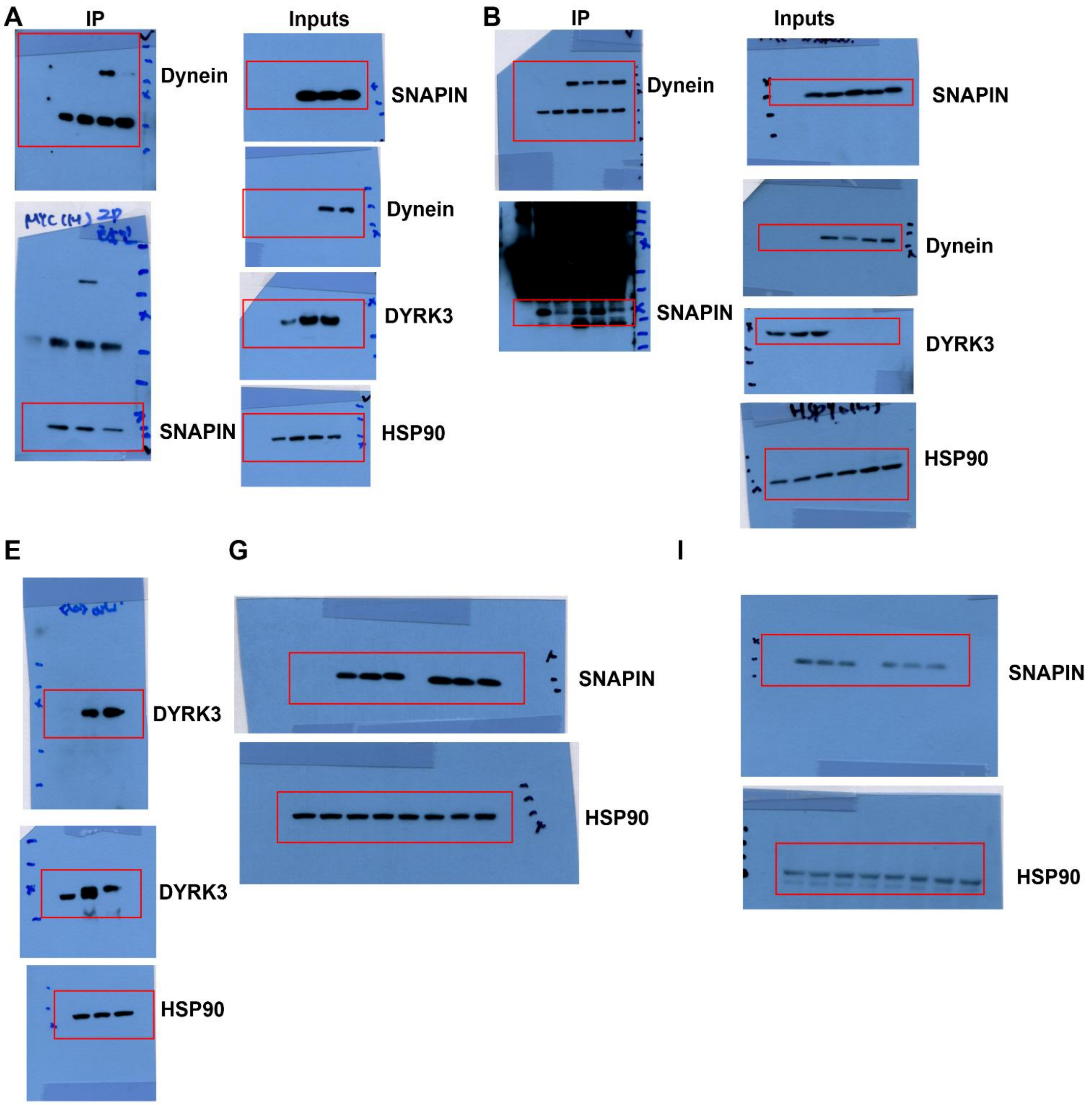

A
IP
Inputs
Dynein
SNAPIN
Dynein
DYRK3
SNAPIN
HSP90
B
IP
Inputs
Dynein
SNAPIN
SNAPIN
Dynein
DYRK3
HSP90
E
DYRK3
DYRK3
HSP90
G
SNAPIN
HSP90
I
SNAPIN
HSP90

Supplementary Figure 1.

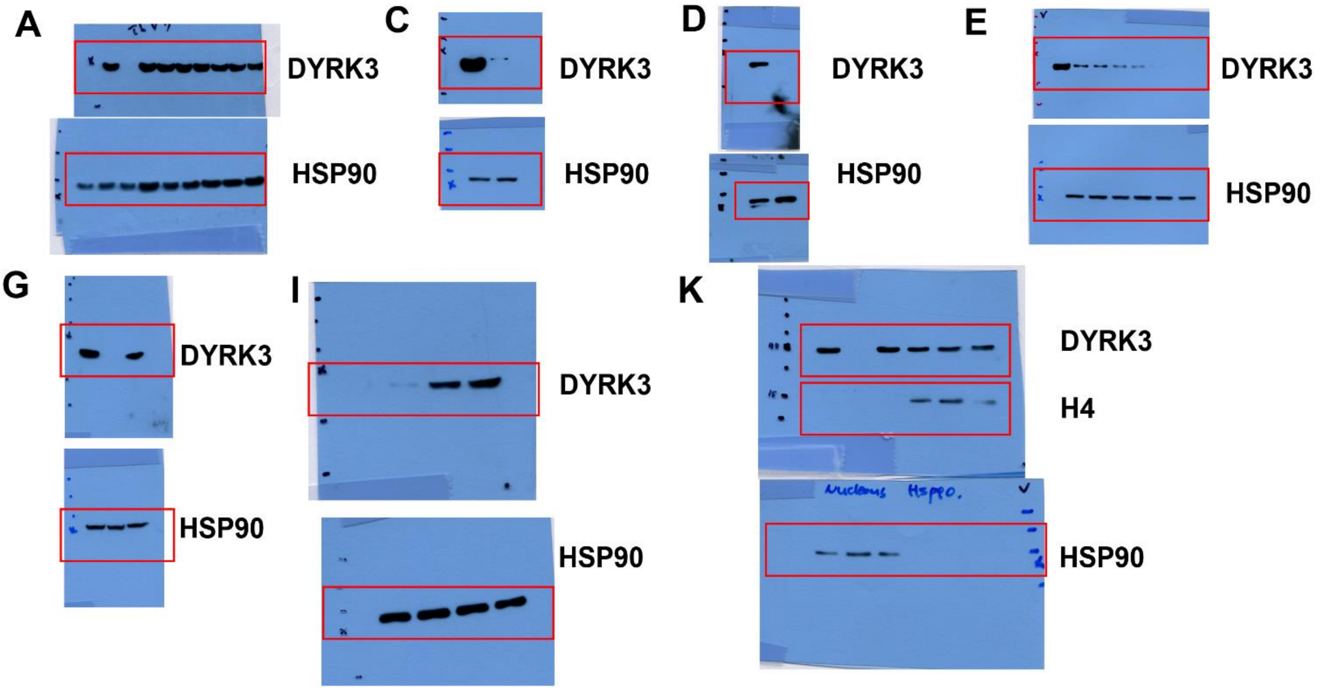

Supplementary Figure 2.

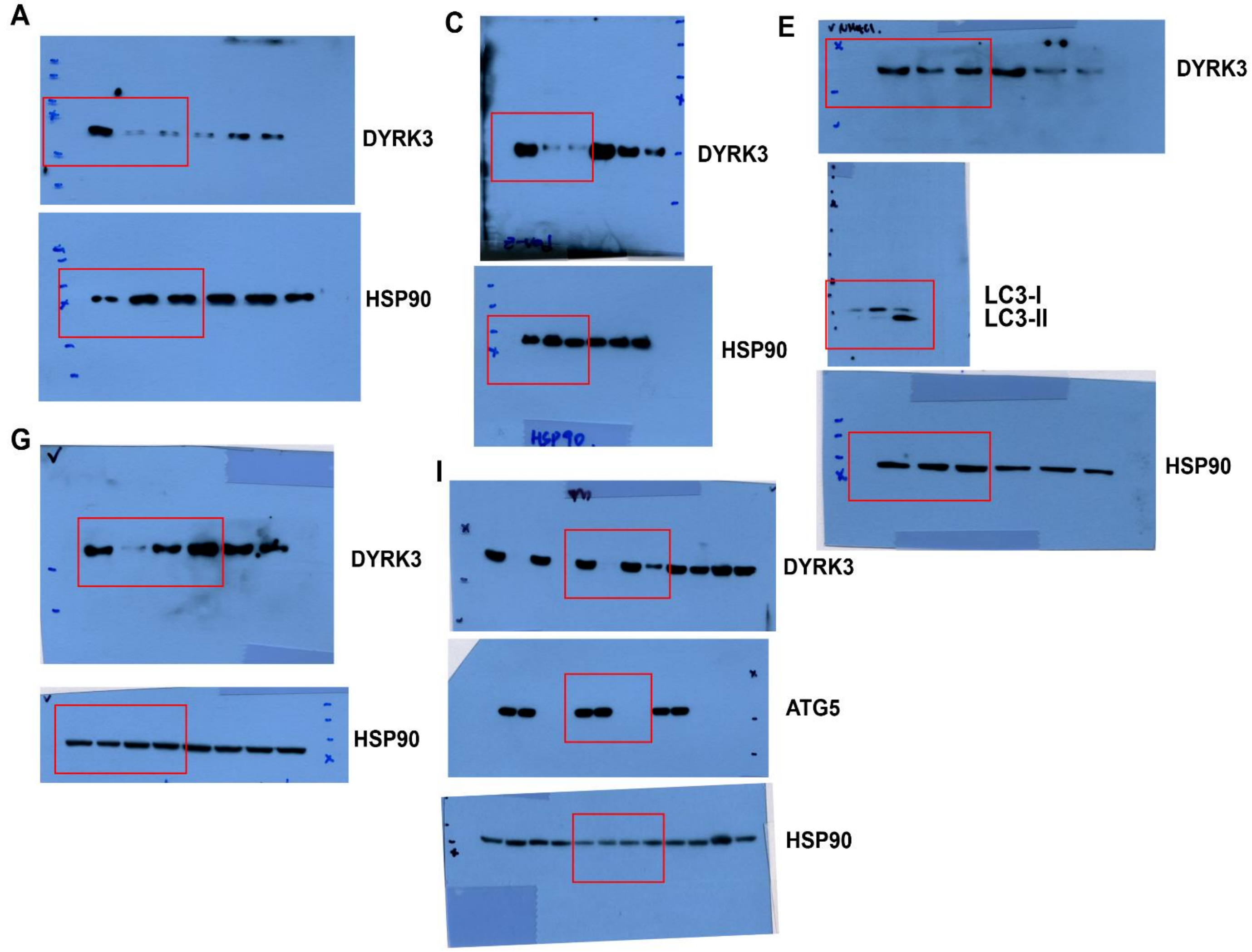

Supplementary Figure 3.

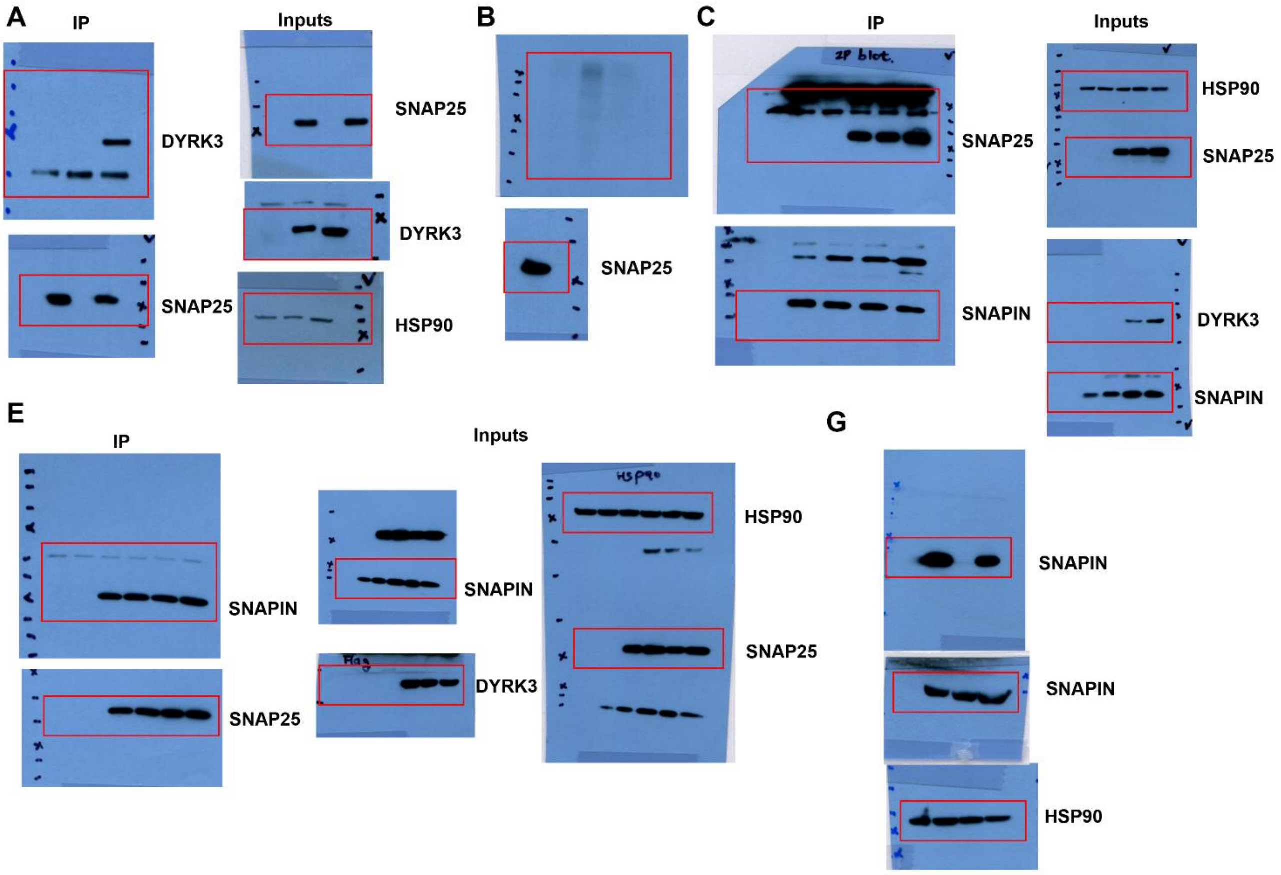

Supplement: Supplementary file 3 — Original Data File [file 41420_2022_1290_MOESM3_ESM.pdf]
